# Prolyl-tRNA synthetase as a novel therapeutic target in multiple myeloma

**DOI:** 10.1038/s41408-023-00787-w

**Published:** 2023-01-12

**Authors:** Keiji Kurata, Anna James-Bott, Mark A. Tye, Leona Yamamoto, Mehmet K. Samur, Yu-Tzu Tai, James Dunford, Catrine Johansson, Filiz Senbabaoglu, Martin Philpott, Charlotte Palmer, Karthik Ramasamy, Sarah Gooding, Mihaela Smilova, Giorgia Gaeta, Manman Guo, John C. Christianson, N. Connor Payne, Kritika Singh, Kubra Karagoz, Matthew E. Stokes, Maria Ortiz, Patrick Hagner, Anjan Thakurta, Adam Cribbs, Ralph Mazitschek, Teru Hideshima, Kenneth C. Anderson, Udo Oppermann

**Affiliations:** 1 Jerome Lipper Multiple Myeloma Center, LeBow Institute for Myeloma Therapeutics, Dana-Farber Cancer Institute, Harvard Medical School, Boston, MA 02215 USA; 2 Botnar Research Centre, Nuffield Department of Orthopaedics, Rheumatology and Musculoskeletal Sciences, University of Oxford, Oxford, OX3 7LD UK; 3 Center for Systems Biology, Massachusetts General Hospital, Boston, MA 02114 USA; 4 Harvard Graduate School of Arts and Sciences, Cambridge, MA 02138 USA; 5 Harvard T.H. Chan School of Public Health, Boston, MA 02115 USA; 6 Department of Biostatistics, Harvard T. H. Chan School of Public Health, Boston, MA 02115 USA; 7 Department of Data Science, Dana-Farber Cancer Institute, Boston, MA 02215 USA; 8 Oxford Centre for Translational Myeloma Research, Botnar Research Centre, University of Oxford, Oxford, OX3 7LD UK; 9 Radcliffe Department of Medicine, University of Oxford, Oxford, OX3 7LD UK; 10 Weatherall Institute of Molecular Medicine, University of Oxford, Oxford, OX3 7LD UK; 11 Department of Chemistry & Chemical Biology, Harvard University, Cambridge, MA 02138 USA; 12 Department of Bioengineering, Northeastern University, Boston, MA 02115 USA; 13 Bristol Myers Squibb, Summit, NJ 07901 USA; 14 Broad Institute of MIT and Harvard, Cambridge, MA 02142 USA

**Keywords:** Myeloma, Drug development

## Abstract

Multiple myeloma (MM) is a plasma cell malignancy characterised by aberrant production of immunoglobulins requiring survival mechanisms to adapt to proteotoxic stress. We here show that glutamyl-prolyl-tRNA synthetase (GluProRS) inhibition constitutes a novel therapeutic target. Genomic data suggest that GluProRS promotes disease progression and is associated with poor prognosis, while downregulation in MM cells triggers apoptosis. We developed NCP26, a novel ATP-competitive ProRS inhibitor that demonstrates significant anti-tumour activity in multiple in vitro and in vivo systems and overcomes metabolic adaptation observed with other inhibitor chemotypes. We demonstrate a complex phenotypic response involving protein quality control mechanisms that centers around the ribosome as an integrating hub. Using systems approaches, we identified multiple downregulated proline-rich motif-containing proteins as downstream effectors. These include CD138, transcription factors such as MYC, and transcription factor 3 (TCF3), which we establish as a novel determinant in MM pathobiology through functional and genomic validation. Our preclinical data therefore provide evidence that blockade of prolyl-aminoacylation evokes a complex pro-apoptotic response beyond the canonical integrated stress response and establish a framework for its evaluation in a clinical setting.

## Introduction

Multiple myeloma (MM), the second most common hematological malignancy, is an incurable cancer of plasma cells [1]. MM cells produce excessive amounts of immunoglobulins and are reliant on protein degradation pathways for survival [2]. Blocking these pathways with proteasome inhibitors (PIs), such as bortezomib (BTZ) or carfilzomib (CFZ), is a common therapeutic strategy that elevates cell stress levels to induce cell death and adversely affects MM pro-survival mechanisms [3, 4]. But while blocking the removal of proteotoxic material is a compelling mechanism to explain how PIs curtail MM [5–7], they also deplete amino acid pools and can be subverted in vitro by the tumour elevating amino acid levels [8].

In fact, recent research [9, 10], including our own [11], suggests that aminoacyl-tRNA synthetase (aaRS) enzymes are attractive therapeutic targets in cancer. Their canonical function is to catalyse the transfer of amino acids to their cognate tRNAs. This process, called “charging”, is highly specific, reliant on ATP, and ensures the continued supply of aminoacyl-tRNAs for protein synthesis. Metabolic changes resulting in amino acid deprivation or inhibition of tRNA charging lead to the accumulation of uncharged tRNAs that bind and activate the general control nonderepressible 2 (GCN2) kinase, a hallmark of the amino acid response (AAR), which in turn, leads to downstream activation of the integrated stress response (ISR) through eIF2α phosphorylation. The ISR is consistently activated at a basal level in MM [12, 13], and targeting this pathway leads to cell death [8, 13].

In mammals, the multi-tRNA synthetase complex (MSC) is a major player in charging tRNA, as it is composed of eight different aaRSs [9]. Among them, human glutamyl-prolyl-tRNA synthetase (GluProRS, gene symbol *EPRS*) is a unique bifunctional aaRS consisting of an N-terminal GST-like domain, a glutamyl-tRNA synthetase (GluRS) domain, a prolyl-tRNA synthetase (ProRS) domain, and a non-catalytic linker WHEP domain that connects the two catalytic domains. Halofuginone (HFG), a febrifugine quinazoline alkaloid derivative, inhibits the ProRS activity of GluProRS in a proline-competitive manner [14]. It triggers G_0_/G_1_ cell cycle arrest, enhances the cytotoxicity of anti-MM agents, and activates several signalling pathways leading to MM cell apoptosis [11]. However, in malaria parasites, ProRS inhibition by HFG is attenuated by elevating proline levels [15], representing a potential resistance mechanism.

In this study, we investigate the role of aaRS in MM using chemogenomic approaches, including a novel pyrazinamide-based ProRS inhibitor, NCP26, that is not affected by proline levels [16]. By investigating this novel inhibitor class in various MM models and data, we pre-clinically validate ProRS as a potential therapeutic target in MM.

## Materials and methods

### Primary patient MM cells, normal B cells, and bone marrow stromal cells (BMSCs)

Blood samples collected from healthy volunteers and bone marrow (BM) aspirates from MM patients were processed by Ficoll-Paque (GE Healthcare Bio-Sciences, Pittsburgh, PA) gradient to obtain mononuclear cells. Normal B cells from healthy volunteers’ peripheral blood were enriched by negative selection methods using EasySep Human B Cell Isolation Kit (STEMCELL Technologies, Vancouver, Canada). For B cell proliferation, isolated B cells were stimulated by 10 μg/mL of human CD40 antibody (R&D systems, Minneapolis, MN, USA) in the presence of 100 U/mL of recombinant human IL4 (R&D Systems). Primary MM cells were further purified by CD138-positive selection using anti-CD138 magnetic-activated cell separation microbeads (Miltenyi Biotec, San Diego, CA). All procedures were performed using a protocol approved by the Institutional Review Board of the DFCI. Informed consent was obtained from all patients and healthy volunteers in accordance with the Declaration of Helsinki.

### Compound synthesis

NCP22, NCP26, ProSA and D-ProSA were synthesised as described [16]. Halofuginol was synthesised as described previously [14, 17].

### AMO1 xenograft model

Five-week-old female CB17 SCID mice (Charles River Laboratories, Cambridge, MA) were subcutaneously injected with 5 × 10^6^ AMO1 cells in a 50/50 mix of culture media/matrigel. Vehicle control or NCP26 (2.5 or 10 mg/kg) was administered once daily via intraperitoneal injection for 21 days. Tumour growth was monitored three times a week using an electronic caliper, and tumour volume was calculated using the formula: (length × width^2^) × 2^−1^, where length is greater than width. All experiments described were approved by and adhered to the guidelines of the Dana Farber Cancer Institute Animal Care and Use Committee.

### Statistical analysis

Experiments were performed independently at least three times, and biological triplicates were used in each experiment unless otherwise specified. Data were analysed using Student *t* tests or χ2 tests for 2 group comparisons or one-way ANOVA followed by Tukey pairwise comparison for multiple comparisons using the Graphpad software (GraphPad Software 9.0.1, La Jolla, CA, USA). (not significant [NS or N]; *P < 0.05; **P < 0.01; ***P < 0.001). Error bars represent standard deviation.

## Results

### Clinical significance and biological role of GluProRS in MM

**Fig. 1:**
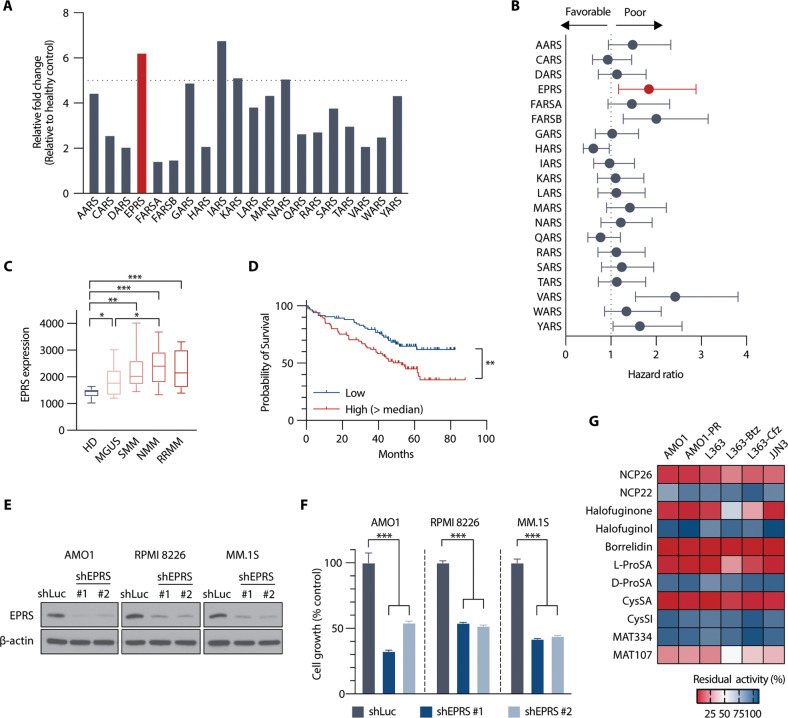
*EPRS* is upregulated and associated with poor prognosis in MM patient studies. **A** Comparative GEP analysis of aminoacyl-tRNA synthetases between normal plasma cells and MM cells (GSE39754). **B** Hazard ratios of aaRS expression levels on survival in MM patients (GSE39754). **C** Comparative GEP analysis of *EPRS* between normal plasma cells and MM cells across disease stages (GSE6477). **D** Overall survival relative to *EPRS* expression in patients with newly diagnosed MM (log-rank test) (MMRF CoMMpass). **E** AMO1, RPMI 8226 and MM.1 S cells were transduced with shLuc (control) or shEPRS (#1, #2). Whole-cell lysates from MM cells were subjected to immunoblotting using indicated antibodies. **F** After puromycin selection, cells were cultured for 48 h, and growth was assessed by MTT assay. Data represent mean ± SD of triplicate cultures. **G** Heatmap of anti-proliferative activities of aaRS inhibitors in MM cell lines – 1 µM (NCP26, NCP22, halofuginone, halofuginol, borrelidin, MAT334 and MAT107) or 5 µM (L-ProSA, D-ProSA, CysSA amd CysSI), 72 h, MTT assay; *n* = 2–5 independent experiments in triplicate technical repeats. HD healthy donor, MGUS monoclonal gammopathy of undetermined significance, SMM smoldering myeloma, NMM newly diagnosed myeloma, RRMM relapsed/refractory myeloma; **P < 0.01, ***P < 0.001.

We found that most aaRSs were upregulated in primary MM cells compared to normal plasma cells from healthy volunteers (microarray dataset of CD138^+^ primary MM cells [GSE39754]) (Fig. 1A). However, only four of the 20 aaRSs correlated with a worse prognosis (Fig. 1B), with only GluProRS (*EPRS*) being both strongly upregulated and associated with poor clinical outcomes. There was a positive correlation between *EPRS* expression and disease progression from monoclonal gammopathy of undetermined significance (MGUS) to therapy refractory MM (Fig. 1C; Supplementary Fig. S1A), and MM patients with high *EPRS* expression levels had significantly shorter survival in two different datasets (GSE39754; *P* = 0.006, MMRF CoMMpass; *P* = 0.007) (Fig. 1D; Supplementary Fig. S1B). *EPRS* is located on chromosome 1q, which is frequently amplified in MM and is a risk factor for MM. We found that *EPRS* gene expression was significantly elevated in patients with a copy number gain in 1q (Supplementary Fig. S1C). Within patients not harbouring a 1q gain/amplification, elevated *EPRS* expression was still associated with inferior progression-free and overall survival (Supplementary Fig. S1D), indicating that *EPRS* expression and 1q amplification are independent risk factors. Furthermore, knocking down *EPRS* using shRNA in three MM cell lines (Fig. 1E; Supplementary Fig. S1E) significantly inhibited cell growth (Fig. 1F), consistent with a key role in maintaining MM cell viability.

To evaluate aaRS inhibition as a possible novel therapeutic strategy, we compiled a focused library of compounds inhibiting distinct aaRS activities. To target the ProRS activity of GluProRS, we used febrifugine derivatives, such as HFG and its analogue halofuginol [14, 17], plus we synthesised and included a series of analogues based on the pyrazinamide scaffold (termed NCP22 in our study) [16, 18]. We also included the polyketide borrelidin, a natural product targeting threonyl-tRNA synthetase (ThrRS) [19], and several amino acyl-adenylate analogues, including D- and L- amino acid analogues (e.g. L-ProSA, D-ProSA), which are unreactive and stable compounds closely mimicking the corresponding amino acyl-AMP intermediates. Significant anti-proliferative effects across all MM cell lines were observed for inhibitors targeting ProRS (NCP26, HFG, ProSA), ThrRS (borrelidin) and CysRS (CysSA) (Fig. 1G). Collectively, these results indicate that several aaRSs, and in particular GluProRS, are critical growth and survival factors in MM cells and can be targeted by small-molecule inhibitors.

### NCP26 is a novel ATP-competitive ProRS inhibitor whose activity is unaffected by increased proline levels

**Fig. 2:**
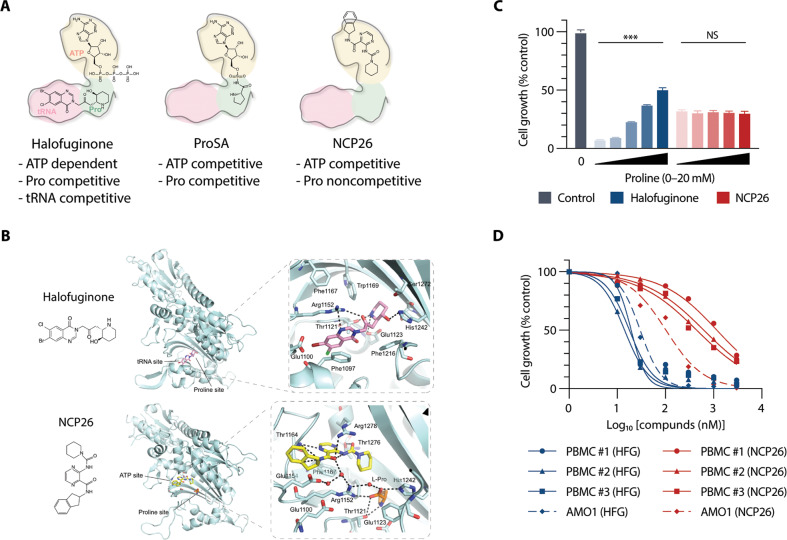
NCP26 is a novel PRS inhibitor chemotype that occupies the ATP pocket. **A** Schematic depiction of PRS inhibitor chemotypes and mode of inhibition: pink—tRNA binding site, light green—proline binding site, olive—ATP binding site. **B** Crystal structures illustrating inhibitor binding modes against human PRS. Inhibitors in complex with human PRS (HFG (PDB ID 4K87; NCP26 PDB ID 7BBU) are shown as coloured stick representations. Key interacting PRS residues are shown in light cyan. **C** AMO1 cells were cultured with or without halofuginone (HFG, 0.5 µM) or NCP26 (0.5 µM) in the presence of proline (0, 1, 5, 10 and 20 mM) for 48 h. Data represent mean ± SD of triplicate cultures. ***P < 0.001. **D** PBMCs isolated from three healthy volunteers were cultured with NCP26 (0.01–10 µM) for 96 h. Data are mean ± SD viability, assessed by MTT assay of triplicate cultures, expressed as percentage of untreated controls.

Having identified ProRS as a possible anti-proliferative target in MM, we further evaluated three ProRS inhibitor chemotypes (Fig. 2A): HFG, ProSA (which is a high-affinity, non-hydrolysable prolyl-adenylate analogue) and NCP26 [16], which we developed based on T-3767758 (NCP22) [18]. Utilising a novel TR-FRET-based biochemical ligand displacement assay, we found that NCP26 inhibited recombinant human ProRS in the presence of 100 μM proline, with a >750-fold increase in affinity (*K*_D_ = 0.35 nM) compared to proline-free conditions (*K*_D_ = 271 nM), which is approximately fivefold more potent than NCP22 in the presence of proline (*K*_D_ = 1.9 nM). The potency of NCP26 is comparable to HFG in the presence of 500 μM ATP (*K*_D_ = 0.19 nM). In the presence of 100 μM proline, HFG showed a >10,000-fold decrease in affinity (*K*_D_ = 2040 nM) [16, 20]. As expected, we also found that human ProRS exhibits a relatively high affinity compared to their endogenous substrate levels (*K*_D, Pro_ = 30.6 μM; *K*_D, ATP_ = 67.1 μM) [16].

To better understand the inhibitor binding modes and to generate a template for future medicinal chemistry campaigns, we determined the crystal structure of NCP26 in complex with human ProRS and proline (Fig. 2B, lower panel) and compared it to HFG binding (Fig. 2B, upper panel). In line with previous structural work on NCP22 [18] and our recent work on NCP26 and Plasmodium ProRS [16], we found that NCP26 does not bind to the proline pocket in human ProRS, but instead to the ProRS ATP binding site, with the 2-aminoindane moiety occupying an additional adjacent pocket. The crystallographic data also suggest that NCP26 is more potent than NCP22 because the piperidinyl ring can adopt a more favourable orientation compared to the energetically unfavourable axial conformation of the NCP22 cyclohexyl moiety (Supplementary Table S1).

Since MM cells and their microenvironment are proline-rich [21–25], we hypothesised that NCP26 would perform better than proline-competitive ProRS inhibitors, such as HFG, in vivo [14]. Therefore, we evaluated the effect of NCP26 vs. HFG in serum-starved AMO1 and RPMI 8226 cells in the presence of proline (Fig. 2C; Supplementary Fig. S1F). As expected, proline abrogated HFG-mediated cell growth inhibition in a dose-dependent manner but did not affect NCP26-mediated cell growth inhibition. We also examined the growth inhibitory effect of NCP26 vs. HFG (0.01–10 µM, for 96 h) in phytohemagglutinin (PHA)-stimulated peripheral blood mononuclear cells (PBMCs) and found a tenfold difference in EC_50_ between PBMCs (EC_50_ ~1 µM) and AMO1 (EC_50_ = 0.1 µM) MM cells for NCP26 but not for HFG at any of the dose ranges (Fig. 2D). Taken together, these results indicate that NCP26 may have a more favourable therapeutic window than HFG and that its anti-proliferative effect is not altered by proline levels in the tumour environment.

### NCP26 is an effective anti-proliferative inhibitor in MM cell lines

**Fig. 3:**
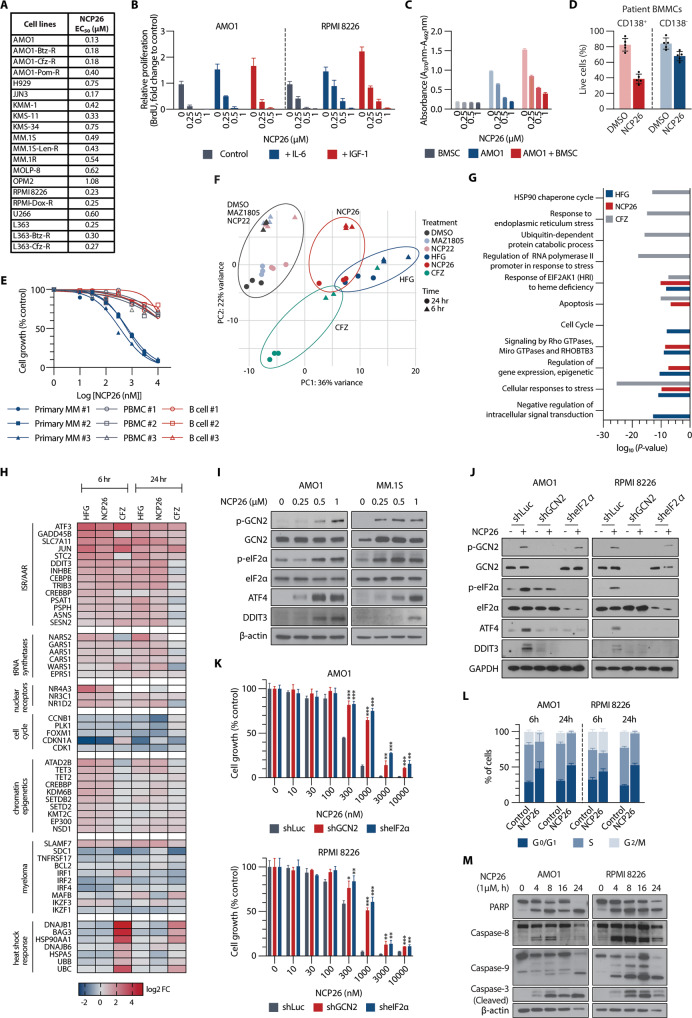
NCP26 exposure induces the integrated stress response via GCN2 and leads to subsequent apoptosis in MM cells. **A** Table of EC_50_ values for NCP26 determined for wild-type and drug-resistant MM cell lines. **B** Serum-starved AMO1 and RPMI 8226 cells were pre-incubated with NCP26 (0.25, 0.5 and 1 µM) for 1 h and then treated with IL-6 (10 ng/ml) or IGF-1 (50 ng/ml). **C** AMO1 and RPMI 8226 cells were cultured with NCP26 (0.25, 0.5 and 1 µM) for 48 h in the presence or absence of BMSC. **D** BMMCs from MM patients were cultured with or without NCP26 (1 µmol/L) for 48 h and analysed using multi-channel flow cytometry. Viability of CD138-positive MM cells and CD138-negative normal BM stromal cells was determined by Annexin V and PI staining. The percentage of viable MM cells from five different patients is shown. **E** Bone marrow CD138^+^ tumour cells from three MM patients and PBMCs and B cells isolated from three healthy volunteers were cultured with NCP26 (0.01–10 µM) for 48 h. **F** PCA plot of transcriptomic (RNAseq) changes in AMO1 cells after a 6- or 24 h exposure with HFG or NCP26 (both 1 µM) or carfilzomib (10 nM). **G** Pathway analysis shows enrichment for stress responses in the RNAseq dataset. **H** Heatmap of selected differentially expressed genes upon NCP26 treatment. **I** Western blot demonstrating dose-dependent responses to NCP26 for canonical ISR activation with concomitant GCN2 and eIF2α phosphorylation. **J**, **K** AMO1 and RPMI 8226 cells were transduced with shLuc (control), shGCN2 or sheIF2α. After puromycin selection, cells were treated with or without NCP26 (0.5 µM) for 6 h (**J**) or 24 h at indicated doses (**K**). Whole-cell lysates from MM cells were subjected to immunoblotting using indicated antibodies (**J**). **L** AMO1 and RPMI 8226 cells were cultured with NCP26 at 0.5 µM for 6 or 24 h. G_0_/G_1_, S and G_2_/M phase in cell cycle profiling was analysed by flow cytometry. Means ± SD from two independent experiments. **M** AMO1 and RPMI 8226 cells were cultured with NCP26 for the indicated times at the indicated dose. Whole-cell lysates were subjected to immunoblotting using indicated antibodies. Cell growth was assessed by MTT assay (**A**, **B**, **E**, **K**) or BrdU uptake (**C**). Data represent mean ± SD of triplicate cultures. **P < 0.01, ***P < 0.001.

We next examined NCP26 in a panel of MM cell lines with major genomic aberrations. NCP26 significantly decreased cell growth with an EC_50_ of ~0.5 µM in most MM cell lines (EC_50_ values between 135 nM in AMO1 and 1.1 µM in OPM2 cells) (Fig. 3A). NCP26 also showed strong anti-proliferative effects against cells resistant to anthracycline (doxorubicin), PIs (BTZ, CFZ) or immunomodulatory drugs (IMiDs; pomalidomide, lenalidomide) (Fig. 3A; Supplementary Fig. S1G). Notably, NCP26 also inhibited cell lines from other hematological malignancies such as leukaemia and lymphoma (Supplementary Fig. S1H).

The cellular components in the BM microenvironment, including stromal cells, can protect MM cells from apoptosis induced by conventional therapeutic agents. Specifically, IL-6 and IGF-1 are secreted factors that promote MM cell growth, survival, and drug resistance in the BM milieu [26]. We therefore first examined whether NCP26 maintains its anti-proliferative effect against MM cells in the presence of exogenous IL-6 or IGF-1 and found that neither impacted NCP26’s effects on the AMO1 and RPMI 8226 cell lines, as assessed by BrdU uptake (Fig. 3B). Next, AMO1 cells were cultured with increasing doses of NCP26 for 24 h in the presence or absence of bone-marrow-derived stromal cells (BMSCs) from MM patients. BMSCs enhanced BrdU incorporation (1.5-fold), which was significantly inhibited by NCP26 (Fig. 3C). NCP26 also induced the apoptosis of CD138^+^ tumour cells from MM patients, with minimal effects on normal components of the BM (Fig. 3D; Supplementary Fig, S1I). In addition, NCP26 induced dose-dependent growth inhibition at EC_50_ values in the range of 350–680 nM in CD138^+^ tumour cells from three MM patients, with a considerably lower inhibitory effect towards PBMCs (EC_50_ 27–36 µM) or CD40 antibody- and IL-4-stimulated B cells (EC_50_ > 20 µM) from healthy volunteers (Fig. 3E). Taken together, these results show that NCP26 treatment is effective in the presence of bone-marrow-derived factors, displays selectivity towards tumour cells over normal cells, and shows significant anti-proliferative activities against a wide spectrum of MM cell lines, including drug-resistant lines.

### NCP26 treatment engages the ISR via GCN2 activation and induces apoptosis

To understand the cellular mechanisms of NCP26, we used RNAseq after a 6 h or 24 h exposure (1 µM) of AMO1 cells to HFG, halofuginol (MAZ1805), NCP22 or NCP26 (Supplementary Data 1). Principal component analysis showed a clear distinction between conditions, with halofuginol and NCP22 clustering with the solvent control (DMSO, 0.1%), consistent with their lower or absent inhibitor potencies (Fig. 3F; Supplementary Table S1). The 24-h treatment of NCP26 and HFG clustered together, along with the 6 h CFZ treatment, due to an overlapping engagement of stress, cell cycle, and apoptosis pathways (Fig. 3G), but they differed in that NCP26 and HFG influenced gene expression and GTPase signalling pathways, while CFZ treatment resulted in a pronounced induction of the heat shock response and ubiquitin-mediated processes, as expected for a PI (Fig. 3H). Importantly, NCP26-treated CFZ-resistant L363 cells similarly showed engagement of the ISR and apoptotic effects (Supplementary Fig. S2A–D), suggesting that targeting ProRS can overcome PI drug resistance.

Inhibition of ProRS increases the concentration of uncharged tRNAs, which would activate the AAR via autophosphorylation of GCN2, triggering the ISR via eIF2α phosphorylation and potentially leading to cell death. Therefore, we next investigated the induction of key ISR elements (GCN2, eIF2α, ATF4, DNA Damage Inducible Transcript 3 (DDIT3)) by Western blotting in AMO1 and MM.1 S cells after exposure to NCP26. As expected, NCP26 increased the phosphorylation of GCN2 and eIF2α, leading to upregulation of ATF4 and DDIT3 (Fig. 3I). Conversely, co-treatment with a GCN2 inhibitor [27] or the ISR inhibitor ISRIB [28] abrogated DDIT3 and ATF4 activation induced by NCP26 (Supplementary Fig. S2E). Furthermore, knockdown of *GCN2* or *eIF2α* abrogated DDIT3 and ATF4 activation induced by NCP26 (Fig. 3J) and, importantly, rescued the growth inhibition by NCP26 in AMO1, RPMI 8226, and MM.1 S cells (Fig. 3K; Supplementary Fig. S2F). These results suggest that NCP26 induces the AAR, which induces sufficient ISR to drive MM cells to dysfunction and cell growth inhibition.

To investigate whether the ISR activation leads to cell death, we next performed cell cycle and apoptosis analyses. NCP26 (0.5 µM, 6 h) treatment induced G_0_/G_1_ cell cycle arrest in both AMO1 and RPMI 8226 cells, which was further enhanced at 24 h (Fig. 3L; Supplementary Fig. S3A). In line with increased sub-G_1_ populations (Supplementary Fig. S3B), we observed apoptotic cell death, evidenced by the appearance of Annexin-V^+^ cells, induced by NCP26 (0.5 μM, 48 h treatment) (Supplementary Fig. S3C). Indeed, NCP26 exposure triggered significantly increased staining of JC-1 monomers in a dose-dependent manner (Supplementary Fig. S3D), indicating that mitochondrial damage is a consequence of NCP26 in MM cells. Immunoblotting (Fig. 3M; Supplementary Fig. S3E) confirmed cleavage of caspases-3, –8, –9 and PARP in both a time- and dose-dependent manner. Collectively, these data strongly suggest that the GCN2-eIF2α-ATF4-DDIT3 axis is a major contributor to apoptotic cell death induced by NCP26 treatment in MM cells.

### NCP26 treatment results in proteomic changes affecting multiple survival factors in AMO1 cells

**Fig. 4:**
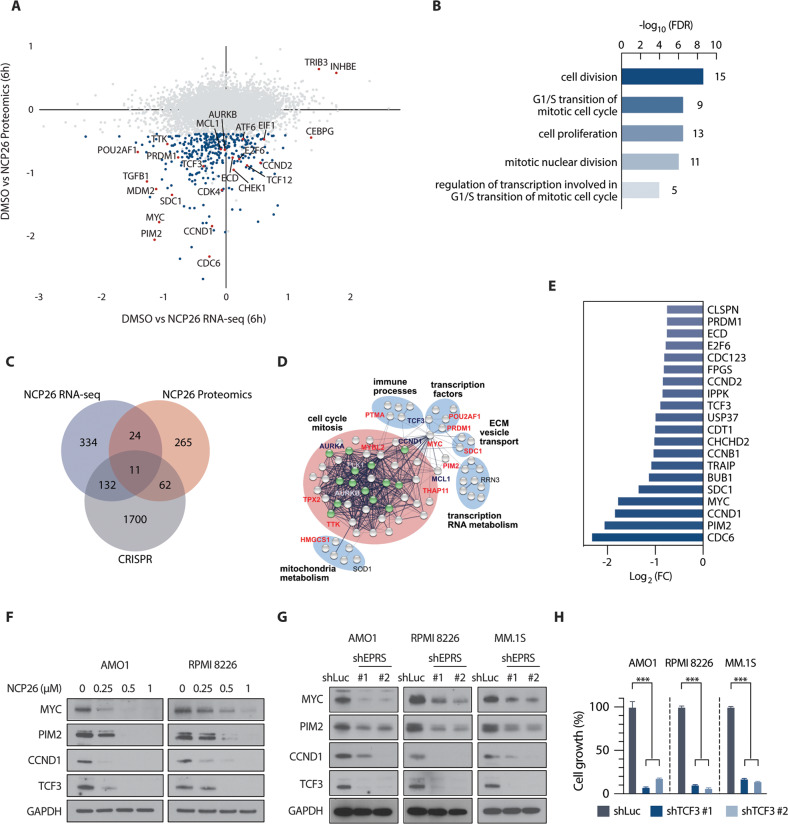
Integration of proteomic, genomic and transcriptomic datasets identifies downstream mechanisms and targets of NCP26 inhibition. **A** Scatterplot of proteomic and RNAseq datasets depicting changes after 6 h of NCP26 exposure in AMO1 cells. Proteins highlighted in blue are proteins downregulated by NCP26, and highlighted in red are discussed in the text (*P* < 0.01). **B** Pathway analysis of downregulated proteins highlights processes related to cell cycle and mitosis. **C** Venn diagram illustrating overlap between downregulated gene transcripts (RNAseq) and proteins (LC/MS proteomics) after 6 h of NCP26 exposure plus CRISPR knockdown targets in MM. **D** STRING analysis of overlapping downregulated proteins (73, intersecting with CRISPR genes and RNAseq (11), or CRISPR (62)) establishes an NCP26 network of essential myeloma mechanisms in AMO1 cells. Cell cycle checkpoints are highlighted in green. Red label: protein overlap from all three datasets. **E** Subset of downregulated proteins upon NCP26 treatment containing proline-rich motifs. **F** AMO1 and RPMI 8226 cells were cultured with NCP26 for 6 h at the indicated doses. Whole-cell lysates were subjected to immunoblotting using indicated antibodies. **G** AMO1, RPMI 8226, and MM.1 S cells were transduced with shLuc or shEPRS (#1, #2) shRNAs. Whole-cell lysates were subjected to immunoblotting using indicated antibodies. **H** TCF3 knockdown results in anti-proliferative activity in MM cells. After puromycin selection of shRNA constructs, cells were cultured for 48 h, and growth was assessed by MTT assay. Data represent mean ± SD of triplicate cultures. ***P < 0.001.

To better understand the mechanistic basis of the NCP26-induced pro-apoptotic effects in MM cells, we correlated proteomic and transcriptomic changes after a 6 h treatment (1 µM) in AMO1 cells. Using tandem mass tag-based quantitative proteomic analysis, we assessed 7366 proteins, of which 7022 proteins and 68,044 unique peptides were quantified at a 1% false discovery rate (Supplementary data). Changes relative to a DMSO control group were determined with a significance level of padj <0.05. Very few proteins had increased abundance (52 proteins with a log2 fold-change between 0.2 and 0.64), with the most abundant proteins being the ATF4 targets TRIB3 (log2FC = 0.64) and INHBE (log2 FC = 0.58), which were also significantly upregulated in the transcriptomic dataset (Fig. 4A). In fact, there was a broad shift towards lower protein abundance compared to the DMSO control, indicating translational stalling as well as ISR activation.

Pathway analysis indicated the downregulated proteins were enriched in cell division, G_1_/S transition of the mitotic cell cycle, cell proliferation, mitotic nuclear division, and regulation of transcription involved in the G_1_/S transition of the mitotic cell cycle (Fig. 4B). Indeed, several key cell cycle regulators, such as CHEK1, CCND1/2, CDK4, CDC6 and ECD, were significantly downregulated at the protein level with minimal changes to their mRNA expression, consistent with a mechanism of translational stalling (Fig. 4A). However, the strong pro-apoptotic effect observed in MM cells suggests that additional pro-survival factors might be downregulated. Indeed, we found several candidates including transcription/chromatin factors, such as MYC, PRDM1, POU2AF1, and survival factors, such as MCL-1 or SDC1 (CD138). A targeted proteomic approach using a mass cytometry panel of myeloma response markers after a 24-h exposure to ProRS inhibitors (Supplementary Fig. S4A and B) supports and extends the observed changes in the protein abundance of MYC, MCL-1 and SDC1.

Several of these also had a significant decrease at the transcriptional level, likely due to a lowered abundance of upstream transcription factors. In agreement with this, we found that several transcription factors, including E2F6, ATF6, TCF12, and CEBPG, were significantly reduced at the protein level without changes in mRNA abundance (Fig. 4A). Importantly, transcription factor binding site analysis, using the ENCODE [29, 30] and JASPAR [31] databases, showed that these transcription factors bind to cis-regulatory elements of *MYC*, *PRDM1*, *POU2AF1*, *SDC1*, and *PIM2* (Supplementary Fig. S5; Supplementary Table S2).

To further identify critical downstream effectors of NCP26-mediated inhibition of MM cell growth, we incorporated genome-wide CRISPR-knockout screening data from the Cancer Dependency Map (DepMap, https://depmap.org/portal/) [32] and correlated these genetic dependencies with the transcriptomic and proteomic datasets from AMO1 cells. We found that 73 proteins downregulated by NCP26 were present in multiple datasets (11 in all 3 datasets, 62 overlapping between CRISPR knockdown and proteomics) (Fig. 4C). STRING network analysis indicated the majority of these are associated with cell cycle progression, but there were others identified including kinases (PIM2, TTK), immune processes (PTMA), metabolic enzymes (HMGCS1), and plasma membrane proteins such as SDC1 (Fig. 4D).

Given that NCP26 blocks the ProRS function, we investigated the abundance of proteins with proline-rich motifs and proline repeats (Supplementary Fig. S6A) and found that proteins with more proline-rich motifs were more downregulated after NCP26 treatment (Supplementary Fig. S6B and C). Interestingly, well-investigated key regulators of MM, such as MYC [33, 34], PIM2 [35], CCND1/2 [33, 36], and aurora kinase A/B (AURKA/B) [37], were listed among the top downregulated proline-rich motifs containing proteins (Fig. 4E). However, we also observed transcripts for several of these candidates (Supplementary Fig. S6D), suggesting a more complex relationship between transcriptomic and proteomic changes upon ProRS inhibition. These NCP26 targets were then validated by Western blot and knockdown experiments (Fig. 4F and G), thus establishing MYC, PIM2, CCND1, and transcription factor 3 (TCF3) as downstream targets of NCP26 inhibition in AMO1 and RPMI 8226 cells.

### TCF3 is downregulated by NCP26 and involved in MM cell proliferation

Above, we identified a network of interacting transcription factors, including MYC, PRDM1, TCF3 and POU2AF1, downregulated by NCP26. While roles for MYC, PRDM1 and POU2AF1 are established in MM [38, 39], we show here that TCF3, a helix-loop-helix transcription factor with a critical role in lymphopoiesis and B cell development [40], is a novel pro-survival factor in MM. First, GEP analyses revealed that tumour cells from MM patients express higher levels of *TCF3* than normal plasma cells, and that *TCF3* expression correlates with disease progression in MM (GSE6477, GSE39754) (Supplementary Fig. S7A and B). Moreover, MM patients with high *TCF3* expression have significantly shorter survival times than patients with lower levels of *TCF3* (GSE39754; *P* = 0.002, MMRF CoMMpass; *P* < 0.001) (Supplementary Fig. S7C and D). This was replicated in an independent refractory/relapse dataset from the CC-4047-MM010 clinical trial (NCT01712789), where inferior survival was correlated with higher *TCF3* expression (Supplementary Fig. S7E and F). TCF3 had reduced expression in the 6 h RNAseq (log2FC = −0.36) and proteomic (log2FC = −0.89) datasets and is an essential gene in the Cancer Dependency Map (Supplementary Fig. S7G). We functionally validated the proliferative role of TCF3 by both pharmacological inhibition of ProRS activity with NCP26 and *EPRS* knockdown (shRNA) (Fig. 4F and G), also including MYC, PIM2 and CCND1 as validation controls. *TCF3* knockdown resulted in markedly decreased TCF3 mRNA and protein levels, associated with significant growth inhibition (Fig. 4H; Supplementary Fig. S7H and I). Collectively, these results suggest that TCF3 mediates MM cell growth and survival.

### NCP26 has anti-tumour activity in a MM xenograft model

**Fig. 5:**
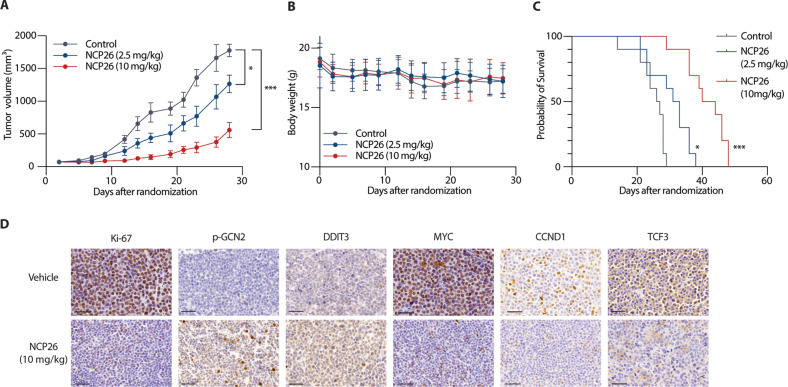
NCP26 reduces tumour burden and increases survival time in human MM xenografts in SCID mice. **A** Mice engrafted with 5 × 10^6^ AMO1 cells were treated intraperitoneally once a day with control vehicle or NCP26 (2.5 mg/kg or 10 mg/kg). Determination of mean tumour volume; error bars represent SD. **B** Body weight change of the mice is shown. **C** Host survival was evaluated from the first day of treatment until death using Kaplan–Meier curves. **D** Immunohistochemistry of selected targets p-GCN2, DDIT3, MYC, CCND1, and TCF3 from day 5 of AMO1 xenograft model. Scale bar = 50 µm. *P < 0.05, ***P < 0.001.

The in vivo efficacy of NCP26 was next evaluated in the AMO1 xenograft mouse model. Mice were inoculated with AMO1 cells (5 × 10^6^ cells) and then randomised into three cohorts (*n* = 10) that received intraperitoneally injected vehicle control, NCP26 at 2.5 mg/kg, or NCP26 at 10 mg/kg once daily for 21 days, in accordance with a preliminary pharmacokinetic study (Supplementary Table S3). NCP26 significantly inhibited AMO1 tumour growth in both treatment cohorts compared with vehicle control (control vs. 2.5 mg/kg, *P* = 0.01; control vs. 10 mg/kg, *P* < 0.001), without significant body weight loss (Fig. 5A,B). Kaplan–Meier curves and log-rank analysis showed a significantly prolonged overall survival in the NCP26 treatment cohort compared to the vehicle control cohort (control vs. 2.5 mg/kg, *P* = 0.01; control vs. 10 mg/kg, *P* < 0.001) (Fig. 5C). Immunohistochemical analyses of harvested tumours confirmed downregulation of NCP26 targets, including MYC, CCND1 and TCF3, and ISR engagement, evidenced by induction of p-GCN2 and DDIT3 (Fig. 5D).

### NCP26 selectively induces the ISR in patient-derived bone marrow MM cells

**Fig. 6:**
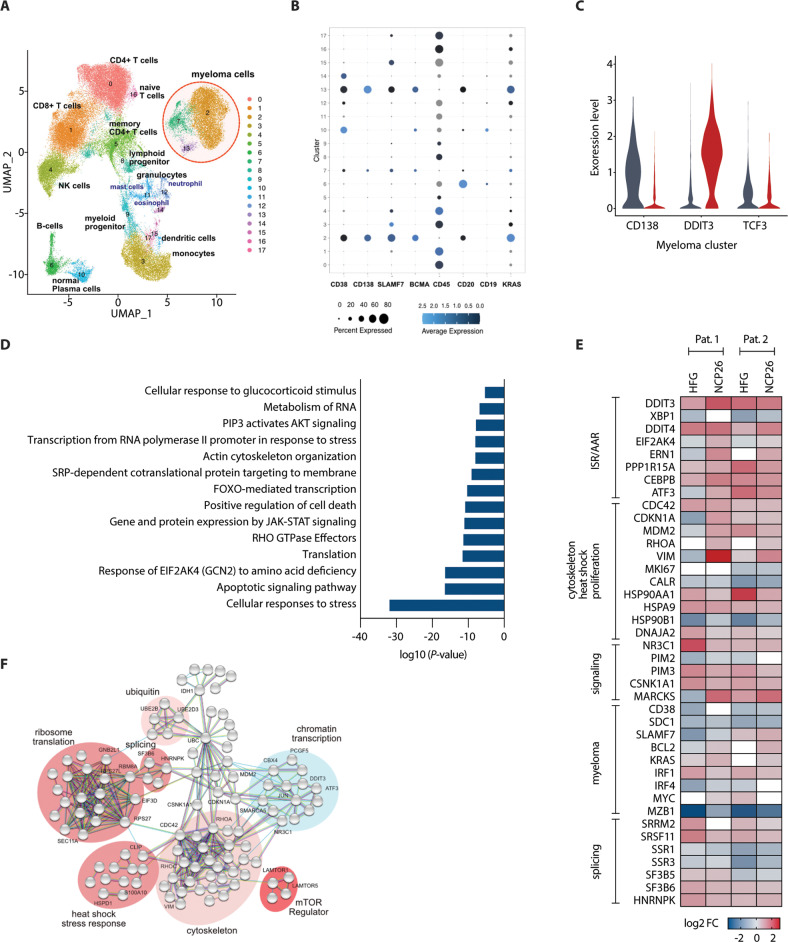
Anti-myeloma activity of NCP26 and HFG in human MM bone marrow assays. **A** UMAP of single-cell RNAseq of human bone marrow samples (newly diagnosed MM), depicting major cell type clusters. **B** Dotplot of selected MM markers and cluster assignment. **C** Violin plot demonstrating the effect of NCP26 exposure (24 h) on myeloma cluster marker CD138 and NCP26 targets DDIT3 and TCF3. **D** Pathway analysis of top 200 regulated genes in myeloma subclusters. **E** Heatmap of selected differentially expressed genes after 24 h exposure with the PRS inhibitors HFG and NCP26. **F** STRING analysis of top regulated genes in the myeloma clusters after 24 h NCP exposure highlighting differentially regulated pathways.

Next, we analysed the effects of ProRS inhibitors in a short-term inhibition (24 h) experiment on BM aspirates from two newly diagnosed MM patients using single-cell transcriptomics as a functional read-out (Supplementary data). Bone marrow mononuclear cells were incubated for 24 h in the presence of 1 µM ProRS inhibitors (HFG and NCP26) or solvent control (DMSO), followed by encapsulation using the Chromium 10× platform, library preparation, and Illumina sequencing. After merging the datasets and filtering, UMAP analysis revealed the expected major immune clusters (Fig. 6A) and 3 distinct myeloma clusters expressing established markers (Fig. 6B). While no significant alterations in most immune cell clusters were noted, myeloid cells responded to treatment with DDIT3 upregulation, indicating ISR activation (Supplementary Fig. S8A). Furthermore, we found downregulation of SDC1 and TCF3 and upregulation of DDIT3 in the myeloma cluster, consistent with the observations in MM cell lines (Fig. 6C).

Pathway analysis of the top 200 differentially regulated genes highlighted the stress response, apoptosis and eIF2α activation as the pathways most significantly affected by ProRS inhibition (Fig. 6D; Supplementary Data). The NCP26 and HFG treatments had similar effects on genes involved in the ISR/AAR, cytoskeleton, cell cycle, proliferation, myeloma marker genes and signalling pathways (Fig. 6E). In addition, NCP26 inhibition affected genes involved in RNA binding and splicing, and in ribosomal processes, further highlighted using a STRING network analysis (Fig. 6F). The relationships identified also emphasise processes in ubiquitin signalling and chromatin, thus underscoring a cascade of complex reactions occurring in MM patient cells, instigated by the canonical ISR upon ProRS inhibition. Importantly, single-cell analysis of a patient with relapsed/refractory MM confirmed these observations by showing a strong induction of DDIT3 and a concomitant SDC1 reduction, associated with a reduction of the myeloma fraction by >50% after 24 h of NCP26 exposure (Supplementary Fig. S8B–E).

## Discussion

We characterised a novel pyrazinamide-based ProRS inhibitor chemotype that induced strong pro-apoptotic responses in myeloma cells. NCP26 does not occupy the proline site in ProRS and is proline-uncompetitive, which is important, since elevated proline levels have been noted in the MM BM [41], possibly driven by MYC and Ras pathways [21, 22, 42]. In addition, we demonstrated that mimicking a shortage of amino acids by blocking the aminoacylation of tRNAs activates GCN2, a hallmark of the AAR, which induced a strong ISR in MM, leading to cell death. This also worked on drug-resistant isogenic cell lines, suggesting that targeting the AAR is a possible therapeutic option for not only addressing proteasome inhibitor resistance but also resistance to other major MM drug classes, such as IMiDs [43].

Our results also shed light on the complex phenotypic consequences of amino acid starvation in MM and point to a central role of ribosome-associated processes during proteasome and ProRS inhibition. In mammalian cells, amino acid stress is sensed at the translating ribosome [44] and at the lysosome [45]. This stress, which can be replicated with aaRS inhibitors, activates GCN2 signalling leading to eIF2α phosphorylation and subsequent attenuation of protein translation [46], which we observe in our data. However, other mechanisms are likely to contribute to the observed pro-apoptotic pattern of phenotypic response. Currently, GCN2 is the only protein in mammalian cells that is known to sense uncharged tRNA [47]. Yet, recent work shows that halofuginone-mediated ProRS inhibition can be sensed and transduced in the absence of GCN2 but requires the presence of the ribosome-associated protein GCN1 [10]. In addition to scaffolding the ribosome to GCN2, GCN1 is proposed to bridge the ribosome to an unknown effector that couples amino acid stress in T cells to downstream regulation of inflammatory and tissue remodelling programs, both of which are not part of the canonical AAR. However, in our work, GCN2 played a central role. In addition, the complexity of ProRS inhibitor responses, the connection to Myc-mediated proliferation and survival, and the regulation of RNA splicing and RNA binding proteins highlight post-transcriptional and translational processes as an important axis to regulate the phenotypic responses to ProRS inhibition.

ProRS inhibition leads to lower levels of prolyl-tRNA and, in our study, to reduced protein expression of proline-rich peptides. However, most peptides were also downregulated at the RNA level, suggesting that transcriptional regulation and ribosomal elongation act in concert, similar to what has been previously observed in a hepatic fibrosis model [48]. Nonetheless, a large proportion of the lower abundance proteins plays a critical role in MM survival and proliferation, including cell cycle genes (such as cyclins, aurora kinases and CDC members [36]), kinases (e.g. PIM2 [49]), transcription factors (MYC [33, 34]) and TCF3, which might cooperate with MYC as an oncogenic axis [50]. Similar to previous work [51], we observed a reduction in the abundance of SDC1, which is an essential survival factor for myeloma that regulates its BM localisation and microenvironment interactions [52].

Although human aaRSs have been considered unsuitable targets owing to their essential role in protein synthesis, studies suggest that normal cells can tolerate aaRS inhibition, in contrast to cancer cells, which require a high amount of protein synthesis. In fact, there are cases of aaRS mutations in hypomyelinating leukodystrophy, where heterozygous carriers for underlying aaRS mutations do not display the disease phenotype [53, 54]. In addition, HFG has therapeutically beneficial effects in fibrotic diseases, such as lung fibrosis or scleroderma [55], based on its ability to reduce the synthesis of proline-rich collagen proteins. HFG does have a narrow therapeutic window with dose-limiting toxicities including nausea, vomiting, fatigue and gastric bleeding [56]. However, there is no evidence that the toxicity results from HFG’s on-target activity or ability to form covalent adducts. HFG analogues that lack this reactivity are better tolerated in animal studies [17].

Taken together, our work indicates that ProRS inhibition is a novel conduit to regulating transcription and mRNA translation of proto-oncogenic factors in MM and subsequent survival, and accordingly represents a novel therapeutic target of interest.

## Supplementary information

Reproducibility checklist

Supplemental Figures S1-S8

Supplemental Methods and Table S1-S6

Supplemental Data S1

Supplemental Data S2

Supplemental Data S3

## Data Availability

Sequencing data have been deposited in the GEO database under accession number GSE186448. Mass spectrometry proteomic data was deposited into the ProteomeXchange Consortium via the PRIDE partner repository with the dataset identifier PXD029453 and 10.6019/PXD029453. Atomic coordinates and structure factors for the human ProRS-NCP26 ligand complex are under the accession number 7BBU with Protein Data Bank (PDB).
