# Correction: Prolyl-tRNA synthetase as a novel therapeutic target in multiple myeloma

**DOI:** 10.1038/s41408-023-00793-y

**Published:** 2023-02-07

**Authors:** Keiji Kurata, Anna James-Bott, Mark A. Tye, Leona Yamamoto, Mehmet K. Samur, Yu-Tzu Tai, James Dunford, Catrine Johansson, Filiz Senbabaoglu, Martin Philpott, Charlotte Palmer, Karthik Ramasamy, Sarah Gooding, Mihaela Smilova, Giorgia Gaeta, Manman Guo, John C. Christianson, N. Connor Payne, Kritika Singh, Kubra Karagoz, Matthew E. Stokes, Maria Ortiz, Patrick Hagner, Anjan Thakurta, Adam Cribbs, Ralph Mazitschek, Teru Hideshima, Kenneth C. Anderson, Udo Oppermann

**Affiliations:** 1 Jerome Lipper Multiple Myeloma Center, LeBow Institute for Myeloma Therapeutics, Dana-Farber Cancer Institute, Harvard Medical School, Boston, MA 02215 USA; 2 Botnar Research Centre, Nuffield Department of Orthopaedics, Rheumatology and Musculoskeletal Sciences, University of Oxford, Oxford, OX3 7LD UK; 3 Center for Systems Biology, Massachusetts General Hospital, Boston, MA 02114 USA; 4 Harvard Graduate School of Arts and Sciences, Cambridge, MA 02138 USA; 5 Harvard T.H. Chan School of Public Health, Boston, MA 02115 USA; 6 Department of Biostatistics, Harvard T. H. Chan School of Public Health, Boston, MA 02115 USA; 7 Department of Data Science, Dana-Farber Cancer Institute, Boston, MA 02215 USA; 8 Oxford Centre for Translational Myeloma Research, Botnar Research Centre, University of Oxford, Oxford, OX3 7LD UK; 9 Radcliffe Department of Medicine, University of Oxford, Oxford, OX3 7LD UK; 10 Weatherall Institute of Molecular Medicine, University of Oxford, Oxford, OX3 7LD UK; 11 Department of Chemistry & Chemical Biology, Harvard University, Cambridge, MA 02138 USA; 12 Department of Bioengineering, Northeastern University, Boston, MA 02115 USA; 13 Bristol Myers Squibb, Summit, NJ 07901 USA; 14 Broad Institute of MIT and Harvard, Cambridge, MA 02142 USA

**Keywords:** Myeloma, Drug development

Correction to: *Blood Cancer Journal* 10.1038/s41408-023-00787-w, published online 12 January 2023

In this article, the author name Anna James-Bott was incorrectly written as Anna-James Bott.

The original article has been corrected.

