# Supplementary material for: Prolyl-tRNA synthetase as a novel therapeutic target in multiple myeloma
Source: Blood Cancer J. 2023 Jan 12;13(1):12. doi: 10.1038/s41408-023-00787-w (PMC9834298; doi:10.1038/s41408-023-00787-w)
Supplement: Supplementary file 2 — Supplemental Figures S1-S8 [file 41408_2023_787_MOESM2_ESM.pdf]

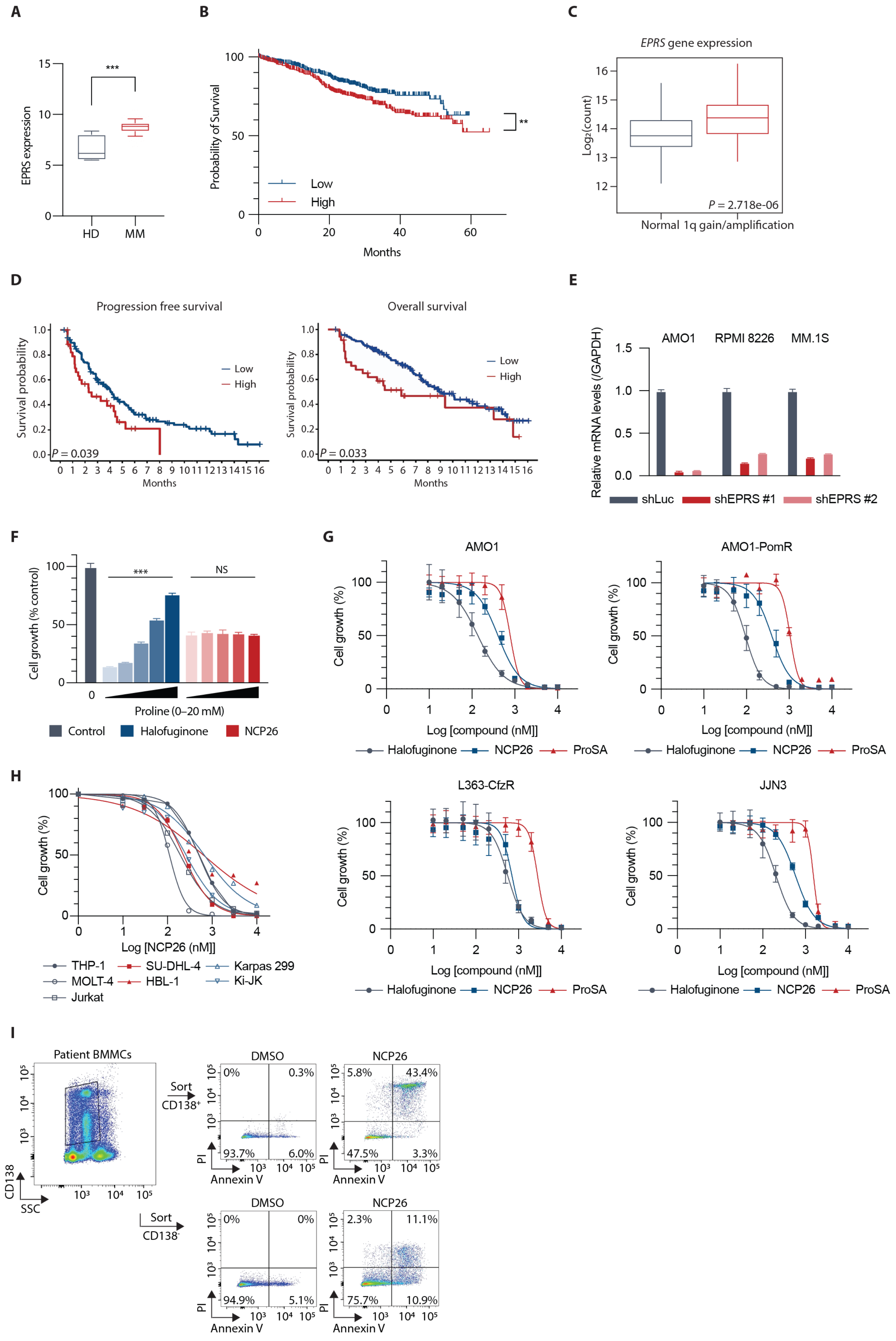

A
EPRS expression
***
HD
MM
B
Probability of Survival
Low
High
Months
**
C
EPRS gene expression
Log2(count)
P = 2.718e-06
Normal
1q gain/amplification
D
Progression free survival
Overall survival
Survival probability
Low
High
P = 0.039
P = 0.033
Months
E
Relative mRNA levels (/GAPDH)
AMO1
RPMI 8226
MM.1S
shLuc
shEPRS #1
shEPRS #2
F
Cell growth (% control)
***
NS
Proline (0–20 mM)
Control
Halofuginone
NCP26
G
AMO1
AMO1-PomR
L363-CfzR
JJN3
Cell growth (%)
Log [compound (nM)]
Halofuginone
NCP26
ProSA
H
Cell growth (%)
Log [NCP26 (nM)]
THP-1
SU-DHL-4
Karpas 299
MOLT-4
HBL-1
Ki-JK
Jurkat
I
Patient BMMCs
CD138
SSC
Sort CD138+
Sort CD138-
DMSO
NCP26
PI
Annexin V
0%
0.3%
93.7%
6.0%
5.8%
43.4%
47.5%
3.3%
0%
0%
94.9%
5.1%
2.3%
11.1%
75.7%
10.9%

Supplementary Fig. S1. ***EPRS*** **expression and prognostic GEP analysis, and antiproliferative activities of aaRS inhibitors.** **(A)** Comparative GEP analysis of *EPRS* between normal plasma cells and MM cells across disease stages (GSE39754). HD, healthy donor; MM, multiple myeloma; RRMM, relapsed/refractory myeloma, ***$P < 0.001$. **(B)** Overall survival relative to *EPRS* expression in patients with MM (log-rank test) (MMRF CoMMpass). **$P < 0.01$, ***$P < 0.01$. **(C)** Comparative GEP analysis of *EPRS* between chromosome 1q normal copy number and 1q gain/amplification from the CC-4047-MM010 clinical trial (NCT01712789). **(D)** Progression free survival (left) and overall survival (right) relative to *EPRS* expression in patients with MM not harboring a 1q gain/amplification (log-rank test) (CC-4047-MM010). **(E)** Quantification of *EPRS* mRNA levels in AMO1, RPMI 8226, and MM.1S cells transduced with either *EPRS*-specific shRNA (shEPRS #1 and #2) or control shRNA targeting *luciferase* (shLuc) by lentivirus. Values represent the amount of mRNA relative to shLuc, which is arbitrarily defined as 1. Data represent mean ± SD of triplicate measurements. **(F)** RPMI 8226 cells were cultured with or without halofuginone (HFG, 0.5 μM) or NCP26 (0.5 μM) in the presence of proline (0, 1, 5, 10, and 20 mM) for 48 h. Data represent mean ± SD of triplicate cultures. ***$P < 0.001$. **(G)** Selected human MM cell lines (AMO1, AMO1 pomalidomide resistant (AMO1-PomR); L363 carfilzomib resistant (L363-Cfz-R), JJN3) were treated for 3 days with increasing concentrations of PRS inhibitor chemotypes (Halofuginone, NCP26 and ProSA). PRS inhibitors HFG and NCP26 are sub-micromolar inhibitors in drug resistant cell lines. Data are mean ± SD viability, assessed by MTT assay of triplicate cultures, expressed as percentage of untreated controls. **(H)** Selected hematological malignancy cell lunes (THP-1, MOLT-4, SU-DHL-4, HBL-1, Jurkat, Karpass 299, and Ki-JK) cultured with NCP26 (0.01-10 μM) for 96 h. Data are mean ± SD viability, assessed by MTT assay of triplicate cultures, expressed as percentage of untreated controls. **(I)** Representative scatter plots of **Fig. 3D** is shown.

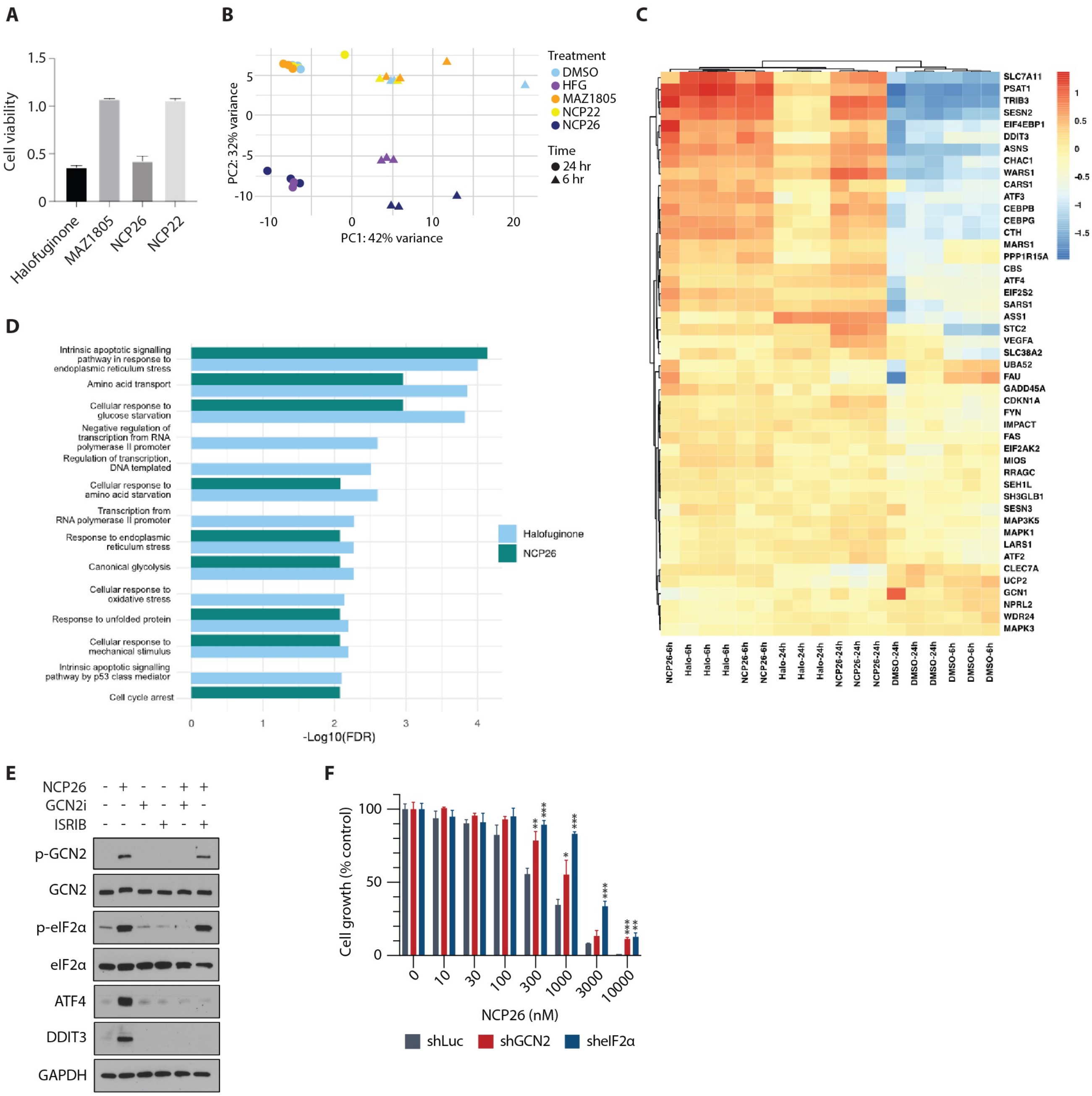

Supplementary Fig. S2. RNA-seq analysis and the involvement of the ISR and GCN2 in ProRS inhibition. (A) CFZ-resistant L363 cells were cultured with NCP22, NCP26, halofuginol (MAZ1805), or HFG (MAZ1392) at 1 μM for 24 h. Data are mean ± SD viability, assessed by MTT assay, expressed as a relative to untreated controls. **(B)** PCA plot of RNA-seq of NCP26 treated CFZ-resistant L363 cells showing separation of conditions. **(C)** Heatmap of normalized expression values for 6 and 24 h treatments on CFZ-resistant L363 cells, demonstrating strong upregulation of ISR genes upon NCP26 and HFG treatment. **(D)** Pathway analysis of halofuginone and NCP26 treated CFZ-resistant L363 cells. **(E)** AMO1 cells were treated with NCP26 (0.5 μM), ISRIB (1 μM) and GCN2 inhibitor (GCN2i, 2 μM) and compound combinations for 6 h. Whole cell lysates from MM cells were subjected to immunoblotting using indicated antibodies. **(F)** MM.1S cells were transduced with shLuc (control), shGCN2 or sheIF2α. After puromycin selection, cells were treated with or without NCP26 (0.5 μM) for 24 h at indicated doses Cell growth was assessed by MTT assay. Data represent mean ± SD of triplicate cultures.

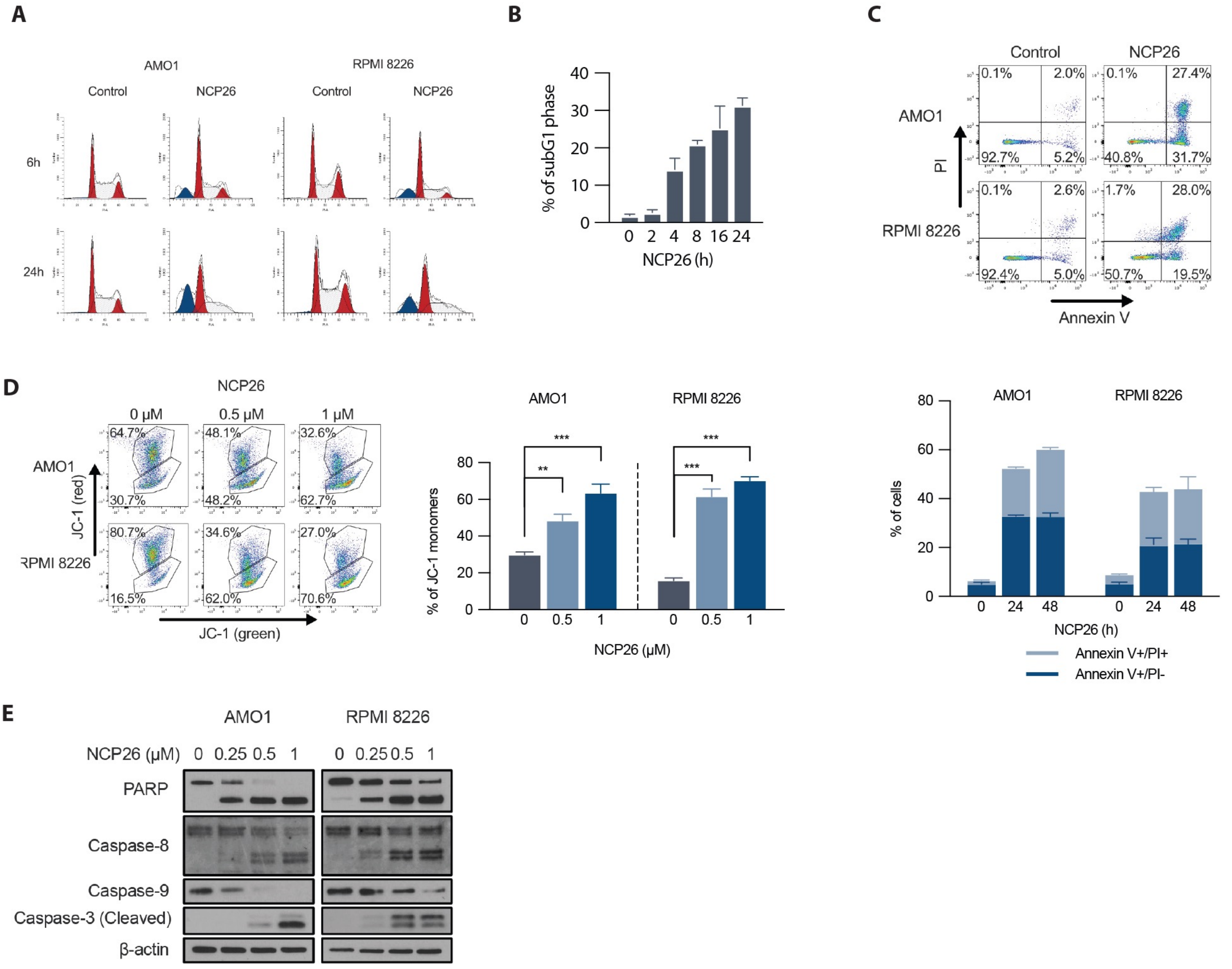

Supplementary Fig. S3. Cell cycle and apoptosis analysis of NCP26 treated MM cells. (A) AMO1 and RPMI 8226 cells were cultured as described in **Fig. 3J**. G_0/G_1, S and G_2/M phase in cell cycle profiling was analysed by flow cytometry. **(B)** AMO1 cells were cultured with NCP26 (0.5 µM) for indicated time periods. The percentage of sub-G0/G1 fraction was analysed by flow cytometry. Means ± SD from three independent experiments. **(C)** AMO1 and RPMI 8226 cells were cultured with NCP26 at 0.5 µM for 48 h. Percentages of early apoptotic (Annexin V^+/PI^-) and late apoptotic (Annexin V^+/PI^+) cells were analyzed by flow cytometry. **(D)** AMO1 and RPMI 8226 cells were treated with NCP26 at 0.5 and 1 µM for 24 h, stained JC-1 dye, and analyzed by flow cytometry. After NCP26 treatment, decreased ΔΨm was indicated by increased JC-1(red)$^-$ population in JC-1(red)$^-$. **(E)** AMO1 and RPMI 8226 cells were cultured with NCP26 for 8 h at the indicated doses. Whole cell lysates were subjected to immunoblotting using indicated antibodies. ***P* < 0.01, ****P* < 0.01

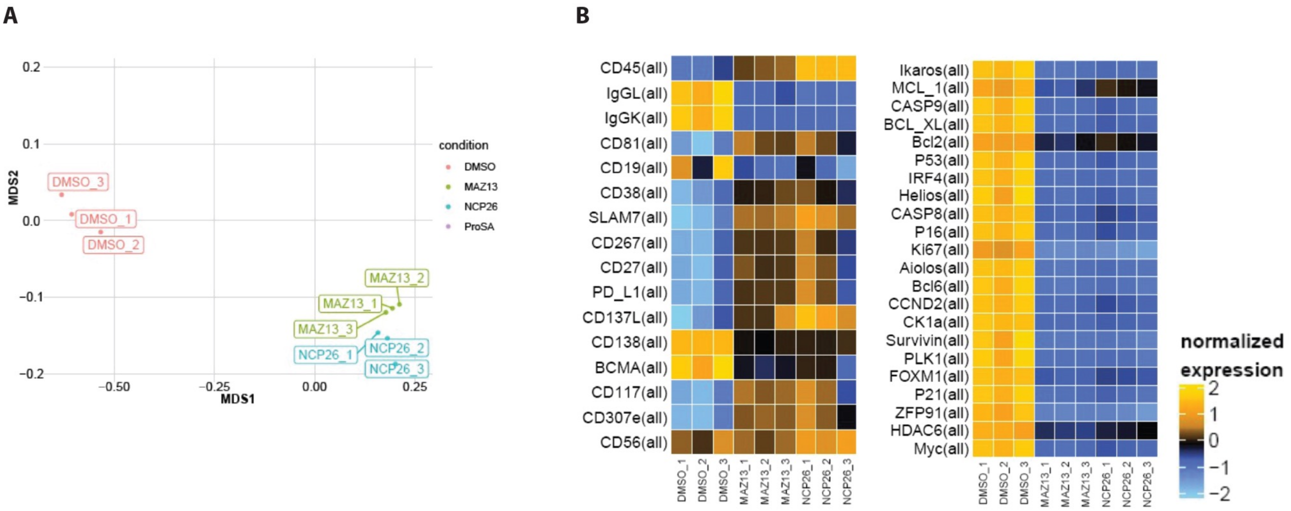

Supplementary Fig. S4. Mass cytometry (CyTOF) in MM cells treated with ProRS inhibitors. (A) AMO1 cells were treated with NCP26 or HFG (= MAZ13) for 24 h in triplicate. Cells were subjected to antibody staining using a panel of metal-tagged antibodies. MDS plot showing separation of conditions, with PRS inhibition by NCP26 and HFG separating from DMSO control. **(B)** Heatmap of MM surface markers (left) and intracellular markers (right) showing significant changes in protein abundances for selected markers upon inhibitor treatments. Whilst all intracellular markers are reduced, a more complex pattern is observed for the surface markers.

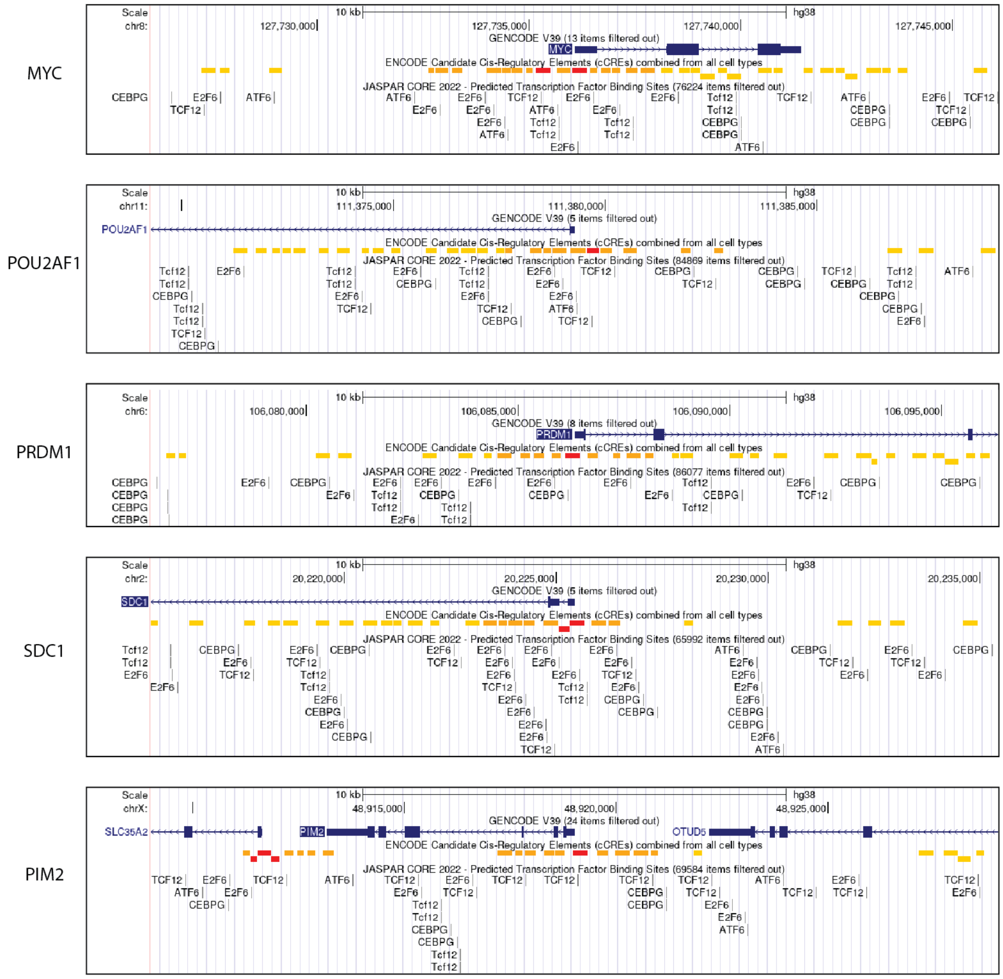

Supplementary Fig. S5. Predicted transcription factor binding sites analysis. Upon identification of candidate cis-regulatory elements (cCREs; promoters and enhancers) using ENCODE database (GRCh38/hg38), the transcription factor binding sites (TFBS) were predicted by the JASPAR database to confirm the DNA sequence of the putative transcription factor binding sites. cCREs and TFBS in MYC, POU2AF1, PRDM1, SDC1 and PIM2 are shown ±10 kb from the Transcription start site. cCREs show promoters (red), proximal enhancers (orange), and distal enhancers (yellow).

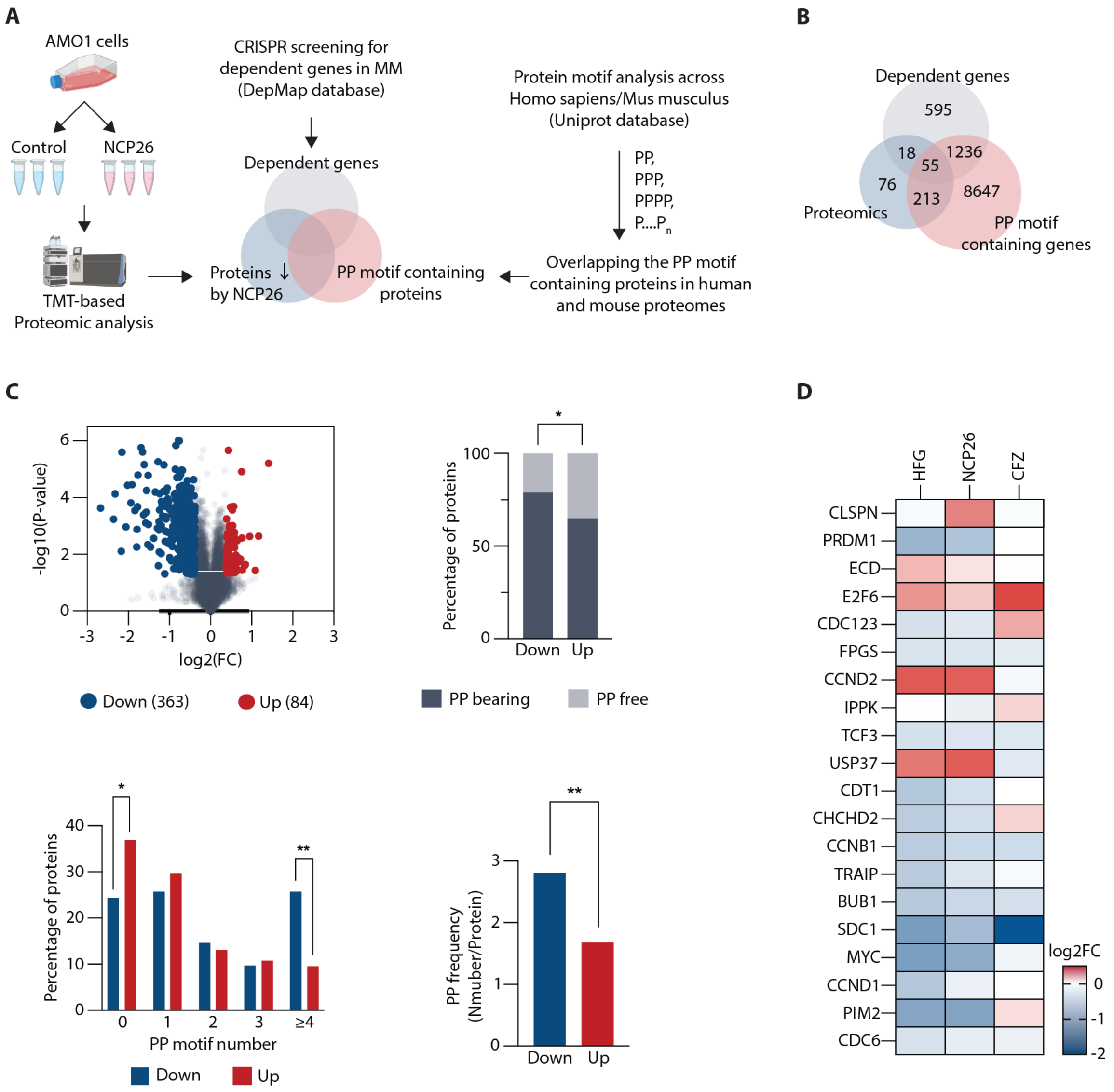

Supplementary Fig. S6. Integrated analysis of proline-rich sequence motifs and abundance in NCP26 treatment. **(A)** Schematic for TMT-based proteomic analysis and *in silico* bioinformatic analysis. **(B)** Integrated genomic and proteomic analyses identify 55 Pro-Pro (PP) motif-containing proteins as preferential downstream targets of EPRS. **(C)** (upper left) Volcano plot depicting the protein level changes induced by NCP26 after 6 h treatment in AMO1 cells. Plots are colored with significant upregulated (red) or downregulated (blue) proteins with fold change (FC) >1.3 and P <0.05. (upper right) PP motif analyses suggest that there are more PP motif-bearing proteins in the downregulated group compared with the upregulated group. (lower left) Distribution of proteins according to the number of PP motifs (0, 1, 2, 3, ≥4) indicate that the downregulated proteins contain more PP motifs compared with upregulated proteins. (lower right) Frequency of PP motif normalized by protein number is significantly higher in the downregulated group compared to the upregulated group. **(D)** Comparison of downregulated Pro-rich proteins in proteomic dataset **(Fig. 4E)** with transcriptomic dataset. Apart from CLSPN, ECD, E2F6, CCND2 and USP37 other Pro-rich transcripts are downregulated in the RNAseq datasets suggesting that changes in transcript levels contribute to the observed lower abundance of Pro-rich proteins in NCP26 treated AMO1 cells.

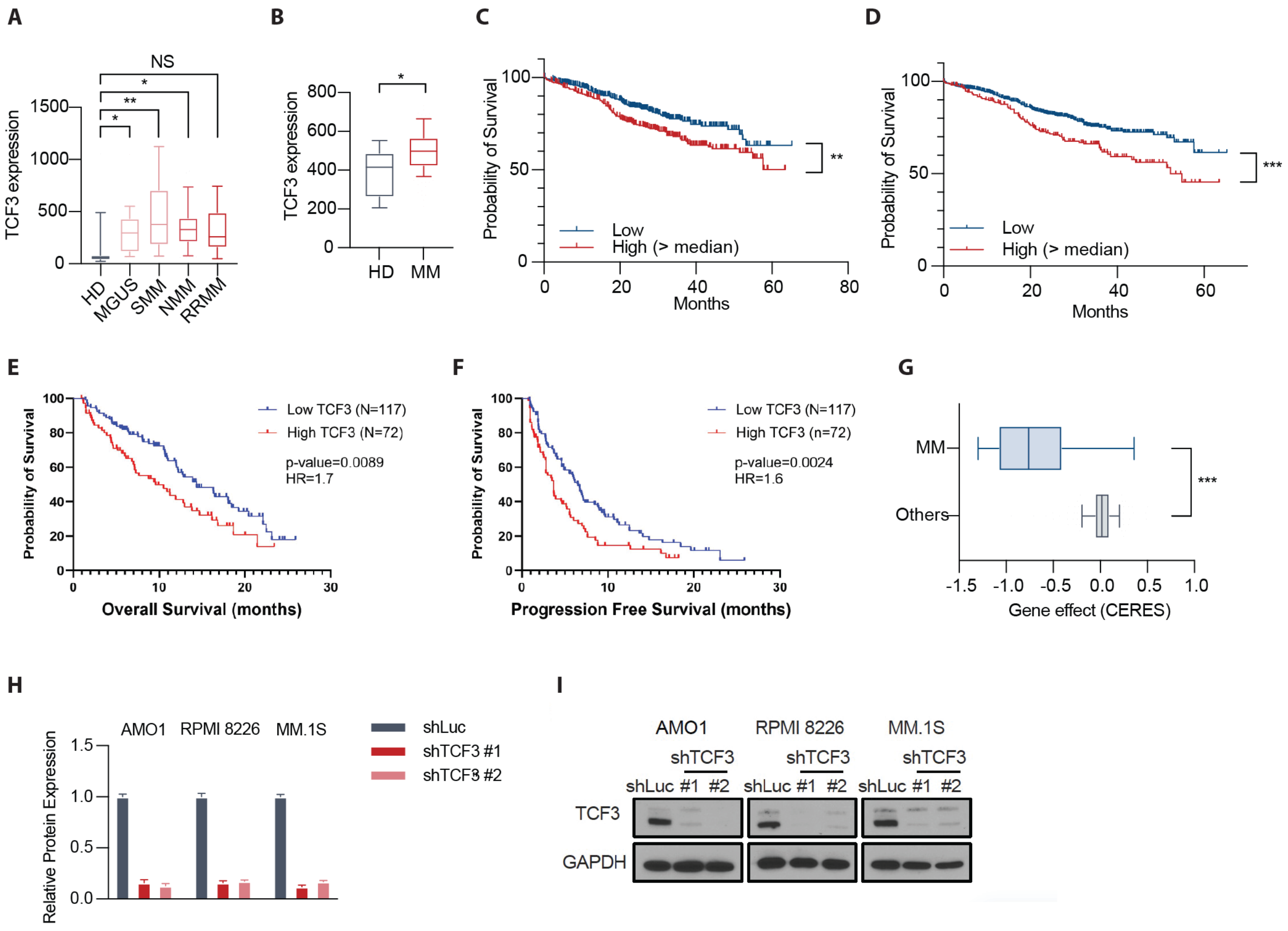

Supplementary Fig. S7. Clinical significance of Transcription factor 3 (TCF3). (A, B) Comparative GEP analysis of *TCF3* between normal plasma cell and MM cells (A; GSE6477, B; GSE39754). **(C, D)** Overall survival relative to *TCF3* expression in patients with newly diagnosed MM (log-rank test) (C; GSE39754, D; MMRF CoMMpass). **(E, F)** Overall survival (E) and progression free survival (F) relative to *TCF3* expression in patients with relapsed/refractory MM (log-rank test) (NCT01712789). **(G)** MM cell lines are more sensitive to TCF3 loss than other cell lines in the CRISPR-Cas9 screening (DepMap). Blue: MM cell lines; Gray: all other cancer cell lines. **(H)** Quantification of *TCF3* mRNA levels in AMO1, RPMI 8226, and MM.1S cells transduced with either *TCF3*-specific shRNA (shTCF3 #1 and #2) or control shRNA targeting *luciferase* (shLuc) by lentivirus. Values represent the amount of mRNA relative to shLuc, which is arbitrarily defined as 1. Data represent mean ± SD of triplicate measurements. **(I)** AMO1, RPMI 8226 and MM.1S cells were transduced with shLuc (control) or shTCF3 (#1, #2). Whole cell lysates from MM cells were subjected to immunoblotting using indicated antibodies.

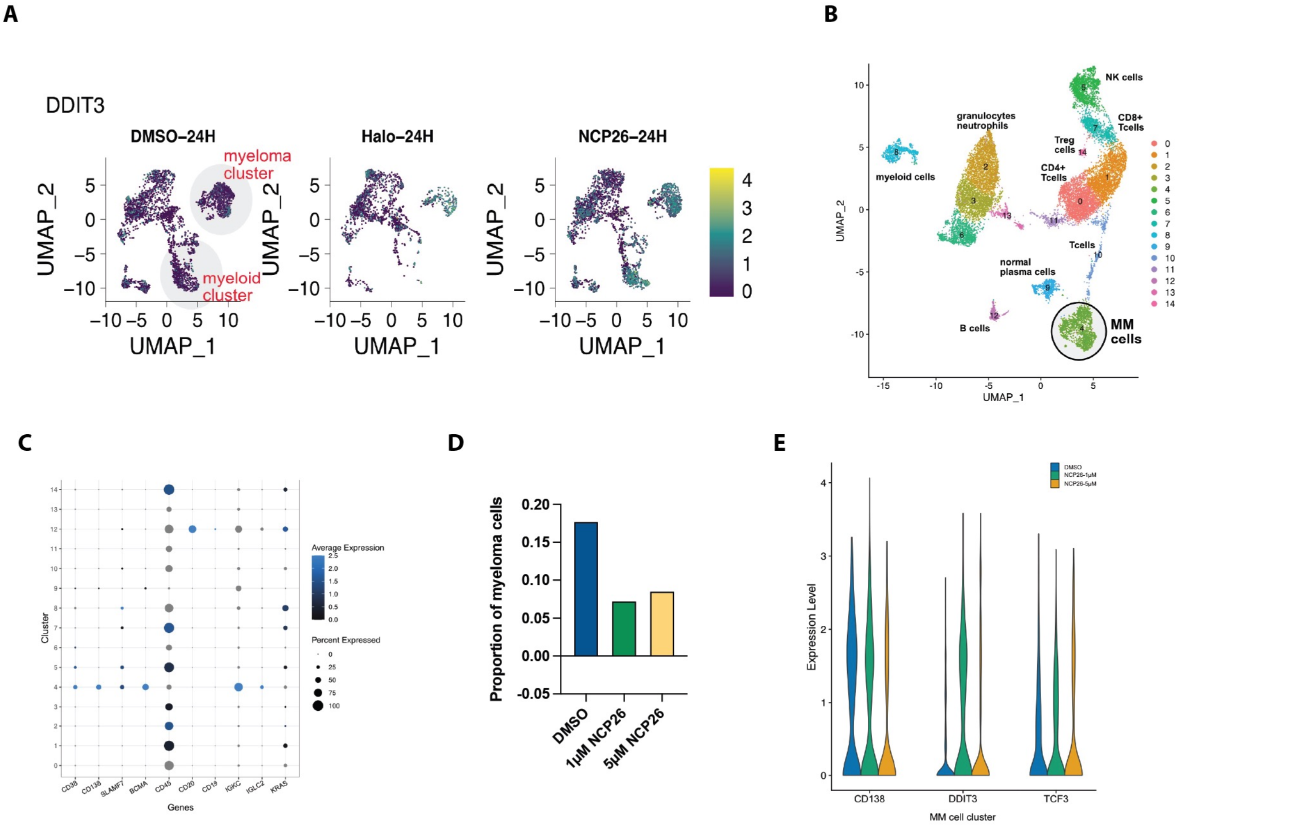

Supplementary Fig. S8. Single-cell transcriptomic analysis with MM patient samples. (A) UMAP (see also Figure 6A main manuscript) of BM clusters and effects of PRS inhibitor treatments (1 µM) Halofuginone (Halo) and NCP26 after 24 h exposure. Expression of ISR response marker DDIT3 is highlighted. HFG reduces cell numbers in the myeloma and myeloid (monocytes, dendritic cells) clusters while NCP26 reduces slightly cell numbers in the myeloma cluster with strong induction of DDIT3 in myeloid and MM clusters. **(B, C)** Bone marrow sample from a relapsed/refractory patient was exposed to NCP26 (1 or 5 µM) for 24 h and single cell analysis was conducted as described using the 10X Genomics Chromium system followed by Illumina sequencing. MM cells were identified. **(D)** The proportion of MM cells was strongly reduced upon 24 h NCP26 exposure. **(E)** Expression analysis shows reduction of CD138 and induction of DDIT3 upon NCP26 exposure in MM cells.
