# Supplementary material for: Prolyl-tRNA synthetase as a novel therapeutic target in multiple myeloma
Source: Blood Cancer J. 2023 Jan 12;13(1):12. doi: 10.1038/s41408-023-00787-w (PMC9834298; doi:10.1038/s41408-023-00787-w)
Supplement: Supplementary file 3 — Supplemental Methods and Table S1-S6 [file 41408_2023_787_MOESM3_ESM.docx]

**Supplemental Methods**

**Cell lines**

The L363, MM.1S, MM.1R, NCI-H929, RPMI 8226, and U266 human MM cells lines, and the THP-1, MOLT-4, and Jurkat human leukemia cell lines, plus the SU-DHL-4 cell line and the HEK293T human embryonic kidney cell line were purchased from American Type Culture Collection (ATCC; Manassas, VA). MOLP-8 cells were purchased from DSMZ (Braunschweig, Germany). KMM-1, KMS-11, KMS-34, and Ki-JK cell lines were obtained from the Japanese Collection of Research Bioresources (National Institute of Health Sciences; Japan). HBL-1 cells were purchased from Applied Biological Materials (Richmond, Canada). Karpass 299 cells were purchased from MilliporeSigma (Burlington, MA). OPM2 cells were provided by Dr. P. Leif Bergsagel (Mayo Clinic, Tucson, AZ). Bortezomib-resistant and carfilzomib-resistant AMO1 cells and lenalidomide-resistant L363 cells were generated in-house. The doxorubicin (Dox)-resistant RPMI-Dox40 cell line was obtained from Dr. William Dalton (Lee Moffitt Cancer Center, Tampa, FL). The lenalidomide-resistant MM.1S cell line was established as previously described [17]. All MM, leukemia, and lymphoma cell lines were cultured in 5% CO_2_ at 37°C in RPMI 1640 medium (Thermo Fisher Scientific, Waltham, MA) containing 10% fetal bovine serum (FBS; Sigma-Aldrich, St Louis, MO), 2 mM L-glutamine, 100 U/mL penicillin, and 100 mg/mL streptomycin (Invitrogen, Carlsbad, CA). HEK293T cells were cultured in Dulbecco Modified Eagle Medium (Thermo Fisher Scientific) containing 10% FBS, 100 U/mL penicillin, and 100 mg/mL streptomycin. Cell lines were tested and authenticated by short tandem repeat DNA fingerprinting analysis (Molecular Diagnostic Laboratory, Dana-Farber Cancer Institute [DFCI], Boston, MA) and used within 3 months after thawing. All cell lines were regularly tested for mycoplasma contamination using the MycoAlert mycoplasma detection kit (Lonza, Basel, Switzerland).

**Compound Screening**

Myeloma cells were plated at 5,000 cells per well in 96-well plates in 90 µl of medium. Compounds were diluted to 10x concentration in media, and 10 µl of compound was added to the wells. Each experiment was performed in triplicate. Cells were grown in the presence of compound for 3, 5 or 7 days as appropriate. Cell viability was assessed using a resazurin oxidation assay by the addition of 10 µl of sodium resazurin (Sigma) (0.01% in PBS) to each well, with an incubation at 37°C for 2 hours followed by reading the fluorescence (Ex 560 nm, Em590 nm) in a plate reader. Drug-resistant cells were screened both in the presence and absence of the appropriate drug. Results from compound-treated wells are expressed as a percentage of the control wells present on each plate. For EC_50_ determination, cells were seeded at 10,000 cells/well into 96-well plates and treated with increasing concentrations of the test compound. Cell viability was detected as described after 5 days. Data was fitted to calculate EC_50_ using Graphpad Prism.

**Cell proliferation, apoptosis, and assessment of mitochondrial membrane potential**

Cell growth was assessed by measuring 3-(4,5-dimethyl- thiazol-2-yl)-2,5-diphenyl tetrasodium bromide (MTT, Chemicon International, Temecula, CA) dye absorbance, as previously described [19]. For drug synergy studies, the combination index was determined using CompuSyn software (ComboSyn Inc) and the Chou–Talalay method [20]. To study the effect of NCP26 on the proliferation of MM cells cultured alone or together with BMSC, MM cells were cultured for 24 h together with BMSC in the presence or absence of NCP26. BMSC were seeded into the 96-well plates 24 h prior to adding MM cells. DNA synthesis was measured with the BrdU cell proliferation enzyme-linked immunosorbent assay (ELISA) kit (Roche Molecular Biochemicals; Mannheim, Germany) using an automated microplate reader, according to the manufacturer's instructions. Apoptosis was evaluated by flow cytometric analysis using the FITC Annexin V Apoptosis Detection Kit II (BD Biosciences, San Jose, CA) per the manufacturer’s instruction. After 24-hour treatment with NCP26, the cells were further incubated with JC-1 (Dojindo, Kumamoto, Japan) at a final concentration of 4 µmol/l at 37°C. The percentage of JC-1-positive cells was determined using a FACS Canto II flow cytometer (Becton Dickinson, San Jose, CA)

**Cell cycle analysis**

MM cells treated with DMSO or NCP26 were harvested, washed with phosphate-buffered saline (PBS), fixed with 70% ethanol, and pre-treated with 10 μg/mL of RNAse (Sigma). Cells were stained with propidium iodide (PI; 5 μg/mL; Sigma), and the cell cycle profile was determined using the FACS Canto II flow cytometer, with analysis using ModFit LT software (Verity Software House, Topsham, ME).

**Cloning, expression, and purification of ProRS**

Human ProRS (construct boundaries Ser1000-Tyr1512) was cloned into pNIC28-Bsa4 with an N-terminal TEV-cleavable histidine tag. The recombinant protein was expressed in a phage-resistant derivative of the Escherichia coli strain BL21(DE3) carrying the pRARE2 plasmid for rare codon expression. Cells were grown at 37°C in Terrific Broth supplemented with either 50 μg/ml kanamycin or 34 μg/ml chloramphenicol, until the culture reached an OD_600_ of 2.0. The temperature was then decreased to 18°C, and protein expression was induced with 0.2 mM IPTG (isopropyl β-D-thiogalactopyranoside) overnight. Cells were collected by centrifugation and resuspended in 50 mM Hepes pH 7.5, 500 mM NaCl, 10 mM Imidazole, 5 % glycerol, 0.5 mM TCEP and a protease inhibitor cocktail (Sigma) and lysed by sonication. The cell lysate was clarified by centrifugation, and the proteins were purified by nickel-affinity chromatography (GE Healthcare) using a stepwise gradient of imidazole. The histidine tag was removed by incubating with TEV protease at 4°C overnight, and this was followed by size exclusion chromatography (Superdex 200, GE Healthcare) in 20 mM Tris pH 8.0, 150 mM NaCl, 5% glycerol, 0.5 mM TCEP and 5 mM MgCl_2_. The TEV protease and the uncleaved proteins were removed by nickel-affinity chromatography and concentrated using an Amicon centrifugal filtration unit. The mass of purified proteins was verified by electrospray ionization time of flight mass spectrometry (ESI-TOF-TOF: Agilent LC/MSD).

**Crystallisation, data collection and structure determination**

The construct encoding human hPRS was incubated with 5 mM L-Pro and 2 mM NCP26 at 39 mg/ml, and then crystallised at 20°C using the sitting drop vapour diffusion method. Crystals were obtained in a drop containing 50 nl of protein compound mixture and 100 nl precipitant consisting of 20% PEG3350 and 0.2 M NH_4_NO_3_ pH 7.5. The crystal was cryo-protected in precipitant solution supplemented with 25% ethylene glycol before it was flash cooled in liquid nitrogen. Data was collected on beamlines I03 at the Diamond Light source UK, and the dataset was processed, scaled and merged at the Diamond light source using Xia2 [21]. An electron density map was obtained by molecular replacement using PHASER, with previously determined structures of hPRS (PDB 4K86) as a search model. The complex structure was solved to 2.19 Å resolution and refined in an iterative process using PHENIX [22] with electron density map inspections and model improvement in COOT [23], and terminated when there were no significant changes in the R_work_ and R_free_ values and inspection of the electron density map suggested that no further corrections or additions were justified ((PDB 7BBU). Structural analysis and figures were done with PyMOL (<http://www.pymol.org>).

**Transcriptomic/Bulk RNA-seq­**

AMO-1 cells at a confluence of 1x10^6^/ml were treated for 6 and 24 hours with 1 µM NCP22, NCP26, MAZ1392 (halofuginone) and MAZ1805 (halofuginol), or DMSO-treated controls. All conditions were performed in triplicate. Approximately 300,000 cells were harvested for each sample for RNA extraction. Cells were centrifuged for 5 minutes at 1500 rpm, the supernatant was removed, and the cell pellets were resuspended in 300 µl of Trizol. 6-hour samples were stored at −80°C overnight, and 24-hour samples were kept on ice prior to RNA extraction. RNA was extracted using Direct-zol RNA Miniprep kits (Zymo Research, USA), following the manufacturer’s instructions. RNA was quantified using a NanoDrop 2000 spectrophotometer and standardised to 100 ng of RNA in 50 µl of nuclease free water. Library prep was performed using Nebnext Ultra II directional RNA library prep kit for Illumina with the Poly(A) mRNA Magnetic Isolation Module (New England Biolabs, USA), following the manufacturer’s instructions. Libraries were quantified using a 2200 Tapestation (Agilent Technologies, USA) with high sensitivity D1000 screen tapes. Libraries were denatured and diluted prior to sequencing. Paired-end sequencing was performed using the Illumina NextSeq 500, according to the manufacturer’s instructions.

**Bulk RNAseq analysis -** FASTQ files were downloaded from Basespace. A cgat-core pipeline within the cgat-flow repository (‘rnaseqdiffexpression’; (<https://github.com/cgat-developers/cgat-flow/blob/master/cgatpipelines/tools/pipeline_rnaseqdiffexpression.py> ) was used to process the reads. Homo sapiens (human) genome assembly GRCh38 (hg38) was used to construct a reference transcriptome. Kallisto [24] was implemented for pseudo-alignment of the reads, with a K-mer size of 31 base pairs. Differential expression analysis was performed using DESeq2 [25]. Pathway analysis was performed using the XGR package [26] and gene ontology (GO) annotations [27].

**PRS inhibition using human BM samples followed by single-cell transcriptomics**

Bone marrow samples were collected from two newly diagnosed multiple myeloma patients. The anonymised human tissue samples used in this project were obtained with informed consent by the HaemBio Tissue Bank (REC reference: 17/SC/0572). After Ficoll gradient separation, mononuclear bone marrow cells were diluted to 500,000 cells/ml in RPMI media supplemented with 2 mM L-glutamine and 10% FBS, and 1 ml was added to 15-ml polypropylene tubes. Compounds were dissolved in DMSO, and 1 mL of compound solution was added to achieve a final concentration of 1 µM and incubated for 24 hours. Cells were counted, and single-cell RNA-seq library preparation was performed using the Chromium Next GEM Single Cell 3' GEM, Library & Gel Bead Kit v3.1, according to the manufacturer’s instructions. Indexed libraries were quantitated by TapeStation, pooled, and sequenced on an Illumina NovaSeq 6000 (Novogene, UK).

**Single-cell RNAseq analysis**

The Scflow pipeline (<https://github.com/Acribbs/scflow)> was used to process scRNA-seq reads. Homo sapiens (human) genome assembly GRCh38 (hg38) was used to construct a reference transcriptome. The Kallisto BUS/ BUStools workflow [28] was implemented to pseudo-align the reads, with a K-mer size of 31 base pairs. The output was converted to single-cell experiment objects and then Seurat objects. Quality control and filtering were performed on the Seurat objects; any cells with a mitochondrial ratio higher than 0.1, fewer than 500 UMIs, or fewer than 300 or greater than 6000 features were removed. Across all samples, the number of cells passing QC and filtering for experiment 1 was 25779, and 61059 cells for experiment 2. Clustering and sample integration was performed using Seurat [29]. To increase granularity, all samples from experiment 1 and experiment 2 were integrated together using Seurat’s SCTransform functionality, using cell-rich samples as references for the integration. Automated cell type annotation was performed using singleR [30], clustifyr [31] and scClassify [32]. The celldex HumanPrimaryCellAtlasData reference annotation was used for singleR and clustifyr. Annotation with ScClassify was performed with the author’s pretrained model ‘Joint Human PBMC’, comprising seven annotated PBMC scRNA-seq data sets. Seurat was used for differential expression analysis.

**Immunoblotting**

Immunoblotting was carried out as in previous studies [33, 34]. Specific antibodies for each protein are listed in Supplementary Table S4.

**Quantitative PCR**

Total RNA was isolated using the RNeasy Mini kit (Qiagen, Hilden, Germany). Complementary DNA was then synthesized from 1 mg of total RNA with oligo(dT) primers using the Superscript III First-strand Synthesis kit (Thermo Fisher Scientific, Waltham, MA). Quantitative real-time polymerase chain reaction (PCR; qRT-PCR) was carried out using Power SYBR Green PCR Master Mix (Thermo Fisher Scientific) and QuantStudio 6 Flex Real-Time PCR System (Applied Biosystems, Foster City, CA). The relative amount of each transcript was determined using the relative standard curve method. The values were normalized to invariant control GAPDH expression. Specific primers for each gene are listed in Supplementary Table S5.

**Lentiviral production and infection for knockdown of specific proteins**

293T packaging cells were seeded into 6-well plates at a density of 8 × 10^5^ cells/well one day prior to transfection. Cells were transfected with 1 µg of transfer plasmid, 1 µg of psPAX2, and 500 ng of pMD2.G for lentivirus packaging, using TransIT-LT1 Transfection reagent (Mirus Bio, Madison, WI, USA), according to the manufacturer’s instructions. After a 16-h transfection, cells were re-fed with fresh medium containing 30% (v/v) FBS to increase viral titer. After 24 h, the medium containing the virus was harvested, passed through 0.45 μm filters, and used immediately for infection. MM cells were spinoculated with crude viral supernatant in the presence of 8 μg/ml polybrene at 1000 g for 60 min at room temperature, followed by additional incubation in 5% CO_2_ at 37°C for 48 h. Infected cells were then selected with puromycin dihydrochloride (Sigma-Aldrich) at 1 μg/ml for at least 2 days and subjected to subsequent assays. All plasmid vectors used in this study are listed in Supplementary Table S6.

**GEP analysis**

Gene Expression Omnibus data sets (GSE39754 and GSE6477), the MM Research Foundation (MMRF) CoMMpass (ClinicalTrials.gov identifier: NCT01454297) and the CC-4047-MM010 clinical trial (ClinicalTrials.gov identifier: NCT01712789) data sets were used for gene expression analyses. The 2456746 or 200843_s_at, and 3845365 or 202648_at were used for *EPRS* and *TCF3*, respectively, on Affymetrix Human Genome U133A Array or Human Exon 1.0 ST Array. Gene expression was estimated with a PLM model. The survival analysis was carried out using the R-package ‘Survival’. Also, ‘surv_cutpoint’ function of the R package “survminer” was used to define the optimal cut-off value for high and low expression of *TCF3* and *EPRS* [35]. Kaplan-Meier survival analysis with the log-rank test was applied to compare the overall survival and progression survival of the patients in the high and low gene expression subgroups.

**Genome-wide CRISPR-knockout screening data analysis**

DepMap (https://depmap.org/portal/) analysis of the dependency of a panel of tumor cell lines on individual genes was conducted using recent (Q1/2021) CRISPR (Avana) Public 20Q4 and Combines RNAi (Broad, Novartis, Marcotte) databases.

**Prediction of transcription factor binding sites on candidate cis-regulatory elements**

Upon identification of candidate cis-regulatory elements (promoters and enhancers) using the ENCODE database (GRCh38/hg38) [36, 37], the transcription factor binding sites were predicted by the JASPAR database [38] to confirm the DNA sequence of the putative transcription factor binding sites. Promoter regions located ±2.5 kb and enhancers located ±10 kb from the transcription start site, respectively, were extracted.

**Immunohistochemical (IHC) analyses**

Following a 5-day treatment with vehicle control or NCP26 (10 mg/kg, once daily), immunohistochemical analysis was performed on the resected tumor from mice, as reported previously [39]. Specific antibodies for each protein are listed in Supplementary Table S4. The primary Abs were visualized with the corresponding biotinylated Ab coupled to streptavidin-peroxidase complex (Vector Laboratories, Burlingame, CA). All Abs, conditions, and reactivity were tested with positive control slides.

**Proteomic analyses and isobaric labelling of peptides**

Cell pellets were mechanically lysed using a mechanical homogenizer with lysis buffer composed of 50 mM HEPES pH 8.5, 250 mM NaCl, and EDTA-free protease inhibitor cocktail (Promega), 5 mM TCEP in 2 % SDS. Lysates were centrifuged at 10,000 x *g* for 10 minutes. Protein content was measured using a BCA assay (Thermo Scientific, Rockford, IL). Disulfide bonds were reduced and cysteine residues alkylated with iodoacetamide (14 mM), as previously described. Protein lysates were purified by methanol-chloroform precipitation, and 200 µg was digested overnight with LysC (Wako, Japan) in a 1/200 enzyme/protein ratio in 2 M urea and 25 mM HEPES, pH 8.5. Digests were acidified with 10% acetic acid (AA) to a pH of ~ 2 and subjected to C18 solid-phase extraction (50 mg SPE) (Sep-Pak, Waters, Milford, MA). Isobaric labeling of the peptides was performed using 10-plex tandem mass tag (TMT) reagents (Thermo Fisher Scientific, Rockford, IL). Reagents (5.0 mg) were dissolved in 252 µl acetonitrile (ACN), and 1/10 of the solution was added to 100 µg of peptides dissolved in 100 µl of 200 mM HEPES, pH 8.5. After 1 hour (RT), the reaction was quenched by adding 3 µl of 5% hydroxylamine. Labeled peptides were combined and acidified prior to C18 SPE on Sep-Pak cartridges (50 mg).

**Basic pH reversed-phase separation (BPRP)**

TMT-labeled peptides were solubilized in 500 μL solution containing 5% ACN/10 mM ammonium bicarbonate, pH 8.0, and 300 µg of TMT-labeled peptides was separated by an Agilent 300 Extend C18 column (3.5 μm particles, 4.6 mm ID and 250 mm in length). An Agilent 1260 binary pump coupled with a photodiode array (PDA) detector (Thermo Scientific) was used to separate the peptides. A 45-minute linear gradient from 10% to 40% acetonitrile in 10 mM ammonium bicarbonate pH 8.0 (flow rate of 0.6 mL/min) separated the peptide mixtures into a total of 96 fractions (36 seconds). The 96 fractions were consolidated into 24 samples in a checkerboard fashion, acidified with 20 µL of 10% formic acid, and vacuum dried to completion. Each sample was desalted via Stage Tips and re-dissolved in 12 µl 5% FA/ 5% ACN, prior to LC-MS/MS analysis.

**Liquid chromatography separation and tandem mass spectrometry (LC-MS/MS)**

Data were collected using an Orbitrap Fusion Lumos mass spectrometer (Thermo Fisher Scientific, San Jose, CA, USA) coupled with a Proxeon EASY-nLC 1200 LC pump (Thermo Fisher Scientific). Peptides were separated on a 75 μm inner diameter microcapillary column packed with 35 cm of GP-18 resin (2.6 μm, 200 Å, Sepax, Newark, DE). For each sample, ~1.5 μg of peptides were separated using a 3 hr gradient of 6–27% acetonitrile in 0.125% formic acid with a flow rate of 400 nL/min. Each analysis used an MS^3^-based TMT method, as described previously [40]. The data were acquired using a mass range of *m/z* 400–1400, resolution 120,000, AGC target 5 x 10^5^, maximum injection time 100 ms, dynamic exclusion of 120 seconds for the peptide measurements in the Orbitrap. Data-dependent MS^2^ spectra were acquired in the ion trap with a normalized collision energy (NCE) set at 35%, AGC target set to 1.8 x 10^4^, and a maximum injection time of 120 ms. MS^3^ scans were acquired in the Orbitrap with an HCD collision energy set to 55%, AGC target set to 1.5 x 10^5^, maximum injection time of 150 ms, resolution at 50,000, and with a maximum synchronous precursor selection (SPS) set to 10.

**Proteomic data analysis**

A compendium of in-house developed software was used to convert mass spectrometric data (Raw file) to the mzXML format, as well as to correct monoisotopic m/z measurements [41]. All experiments used the Human UniProt database (downloaded April 2018), where reversed protein sequences and known contaminants, such as human keratins, were appended. SEQUEST searches were performed using a 20 ppm precursor ion tolerance, while requiring the peptide amino/carboxy (N/C) terminus to have trypsin protease specificity and allowing up to two missed cleavages. Ten-plex TMT tags on peptide N termini and lysine residues (+ 229.162932 Da) and carbamidomethylation of cysteine residues (+57.02146 Da) were set as static modifications, while methionine oxidation (+ 15.99492 Da) was set as variable modification. An MS spectra assignment false discovery rate (FDR) of less than 1% was achieved by applying the target-decoy database search strategy [41]. Filtering was performed using an in-house linear discrimination analysis (LDA) method to create one combined filter parameter from the following peptide ion and MS spectra metrics: SEQUEST parameters XCorr and ΔCn, peptide ion mass accuracy and charge state, in-solution charge of peptide, peptide length, and mis-cleavages. Linear discrimination scores were used to assign probabilities to each MS spectrum for being assigned correctly, and these probabilities were further used to filter the dataset with an MS spectra assignment FDR smaller than 1% at the protein level [42]. For quantification, a 0.003 *m/z* window centered on the theoretical m/z value of each of the six reporter ions and the intensity of the signal closest to the theoretical m/z value was recorded. Reporter ion intensities were further de-normalized based on their ion accumulation time for each MS spectrum and adjusted based on the overlap of isotopic envelopes of all reporter ions (as determined by the manufacturer). The total signal intensity across all peptides quantified was summed for each TMT channel, and all intensity values were adjusted to account for potentially uneven TMT labeling and/or sample handling variance.

**Supplementary Table S1. aaRS inhibitor library**

| Compound | HsProRS (KD [nM]) | | | | | | | | |
| --- | --- | --- | --- | --- | --- | --- | --- | --- | --- |
|  | - | | | + 100 μM proline | | | + 500 μM ATP | | |
|  | mean | lower  95% CI limit | upper  95% CI limit | mean | lower  95% CI limit | upper  95% CI limit | mean | lower  95% CI limit | upper  95% CI limit |
| Halofuginone | 1,160 | 1,032 | 1,302 | 2,044 | 1,748 | 2,415 | 0.225 | 0.186 | 0.272 |
| Halofuginol | > 8.72 μM (IC50 > 10 μM; 77.2% signal at 10 μM) |  |  | > 5.09 μM (IC50 > 10 μM; 85.2% signal at 10 μM) |  |  | 4.67 | 3.91 | 5.58 |
| L-ProSA | 0.0702 | 0.0501 | 0.1017 | < 1 nM |  |  | < 1 nM |  |  |
| D-ProSA | 594 | 555 | 636 | 360 | 318 | 407 | - | - | - |
| NCP22 | 371 | 344 | 401 | 2.16 | 1.92 | 2.43 | 954 | 705 | 1,320 |
| NCP26 | 271 | 250 | 293 | 0.351 | 0.304 | 0.406 | 678 | 425 | 1,160 |

**Supplementary Table S2. Predicted transcription factor binding sites in candidate cis-regulatory elements**

| TF | Matrix ID | Motif sequence | Target  gene | TFBS | ENCODE accession | cCRE  position | ENCODE  label |
| --- | --- | --- | --- | --- | --- | --- | --- |
| E2F6 | MA0471.2 | 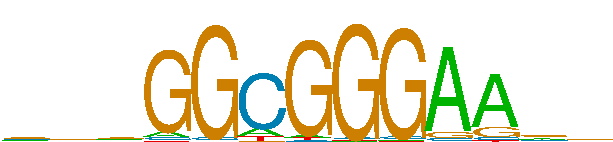 | MYC | chr8:127732908-127732920 | EH38E2666161 | chr8:127732794-127733106 | pELS |
|  |  |  |  | chr8:127734171-127734183 | EH38E2666163 | chr8:127734011-127734357 | pELS |
|  |  |  |  | chr8:127734427-127734439 | EH38E2666164 | chr8:127734366-127734569 | pELS |
|  |  |  |  | chr8:127736163-127736175 | EH38E2666169 | chr8:127736036-127736381 | PLS |
|  |  |  |  | chr8:127736534-127736546 | EH38E2666170 | chr8:127736447-127736616 | pELS |
|  |  |  |  | chr8:127737143-127737155 | EH38E2666172 | chr8:127736966-127737170 | pELS |
|  |  |  | SDC1 | chr2:20217792-20217804 | EH38E1978361 | chr2:20217642-20217903 | dELS |
|  |  |  |  | chr2:20219346-20219358 | EH38E1978364 | chr2:20219337-20219640 | dELS |
|  |  |  |  | chr2:20219921-20219933 | EH38E1978365 | chr2:20219873-20220208 | dELS |
|  |  |  |  | chr2:20220070-20220082 | EH38E1978365 | chr2:20219873-20220208 | dELS |
|  |  |  |  | chr2:20223784-20223796 | EH38E1978373 | chr2:20223659-20223833 | pELS |
|  |  |  |  | chr2:20223967-20223979 | EH38E1978374 | chr2:20223878-20224203 | pELS |
|  |  |  |  | chr2:20224004-20224016 | EH38E1978374 | chr2:20223878-20224203 | pELS |
|  |  |  |  | chr2:20224283-20224295 | EH38E1978375 | chr2:20224231-20224500 | pELS |
|  |  |  |  | chr2:20224483-20224495 | EH38E1978375 | chr2:20224231-20224500 | pELS |
|  |  |  |  | chr2:20224761-20224773 | EH38E1978376 | chr2:20224697-20225042 | pELS |
|  |  |  |  | chr2:20224772-20224784 | EH38E1978376 | chr2:20224697-20225042 | pELS |
|  |  |  |  | chr2:20224880-20224892 | EH38E1978376 | chr2:20224697-20225042 | pELS |
|  |  |  |  | chr2:20225068-20225080 | EH38E1978377 | chr2:20225060-20225314 | PLS |
|  |  |  |  | chr2:20225544-20225556 | EH38E1978378 | chr2:20225328-20225677 | PLS |
|  |  |  |  | chr2:20226420-20226432 | EH38E1978380 | chr2:20226238-20226526 | pELS |
|  |  |  | POU2AF1 | chr11:111371460-111371472 | EH38E1569170 | chr11:111371226-111371558 | dELS |
|  |  |  |  | chr11:111378800-111378812 | EH38E1569188 | chr11:111378760-111379098 | pELS |
|  |  |  |  | chr11:111378995-111379007 | EH38E1569188 | chr11:111378760-111379098 | pELS |
|  |  |  |  | chr11:111379312-111379324 | EH38E1569189 | chr11:111379181-111379530 | pELS |
|  |  |  |  | chr11:111387537-111387549 | EH38E1569197 | chr11:111387393-111387740 | dELS |
|  |  |  | PIM2 | chrX:48928592-48928604 | EH38E2754235 | chrX:48928510-48928693 | dELS |
|  |  |  | PRDM1 | chr6:106085858-106085870 | EH38E2490436 | chr6:106085803-106086007 | pELS |
|  |  |  |  | chr6:106087667-106087679 | EH38E2490441 | chr6:106087563-106087892 | pELS |
|  |  |  |  | chr6:106088629-106088641 | EH38E2490443 | chr6:106088635-106088792 | dELS |
| TCF12 | MA1648.1 | 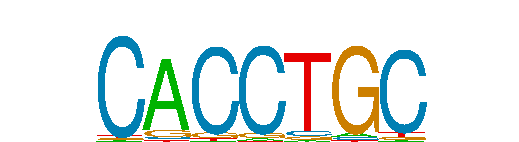 | MYC | chr8:127727336-127727346 | EH38E2666157 | chr8:127727272-127727618 | dELS |
|  |  |  |  | chr8:127735297-127735307 | EH38E2666167 | chr8:127735172-127735514 | PLS |
|  |  |  |  | chr8:127735715-127735726 | EH38E2666168 | chr8:127735668-127735990 | pELS |
|  |  |  |  | chr8:127737457-127737468 | EH38E2666173 | chr8:127737306-127737579 | pELS |
|  |  |  |  | chr8:127739892-127739903 | EH38E2666180 | chr8:127739692-127740013 | dELS |
|  |  |  |  | chr8:127741655-127741665 | EH38E2666183 | chr8:127741497-127741708 | dELS |
|  |  |  | SDC1 | chr2:20217834-20217844 | EH38E1978361 | chr2:20217642-20217903 | dELS |
|  |  |  |  | chr2:20219419-20219429 | EH38E1978364 | chr2:20219337-20219640 | dELS |
|  |  |  |  | chr2:20219635-20219646 | EH38E1978364 | chr2:20219337-20219640 | dELS |
|  |  |  |  | chr2:20224042-20224052 | EH38E1978374 | chr2:20223878-20224203 | pELS |
|  |  |  |  | chr2:20224958-20224968 | EH38E1978376 | chr2:20224697-20225042 | pELS |
|  |  |  | POU2AF1 | chr11:111377229-111377240 | EH38E1569183 | chr11:111376976-111377240 | dELS |
|  |  |  |  | chr11:111377769-111377779 | EH38E1569185 | chr11:111377642-111377798 | pELS |
|  |  |  |  | chr11:111379684-111379694 | EH38E1569190 | chr11:111379572-111379871 | PLS |
|  |  |  |  | chr11:111382604-111382614 | EH38E1569194 | chr11:111382569-111382780 | pELS |
|  |  |  | PIM2 | chrX:48917860-48917870 | EH38E2754221 | chrX:48917856-48918104 | pELS |
|  |  |  |  | chrX:48919102-48919112 | EH38E2754224 | chrX:48918980-48919329 | PLS |
|  |  |  |  | chrX:48920871-48920881 | EH38E2754228 | chrX:48920818-48920997 | pELS |
|  |  |  |  | chrX:48928550-48928560 | EH38E2754235 | chrX:48928510-48928693 | dELS |
|  |  |  | PRDM1 | chr6:106083865-106083876 | EH38E2490431 | chr6:106083606-106083935 | dELS |
|  |  |  |  | chr6:106092377-106092387 | EH38E2490450 | chr6:106092238-106092564 | dELS |
| CEBPG | MA1636.1 | 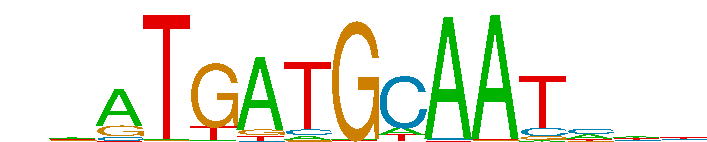 | MYC | chr8:127743506-127743515 | EH38E2666188 | chr8:127743338-127743538 | dELS |
|  |  |  | SDC1 | chr2:20219991-20220005 | EH38E1978365 | chr2:20219873-20220208 | dELS |
|  |  |  |  | chr2:20220588-20220602 | EH38E1978366 | chr2:20220455-20220775 | dELS |
|  |  |  |  | chr2:20219991-20220005 | EH38E1978365 | chr2:20219873-20220208 | dELS |
|  |  |  |  | chr2:20220588-20220602 | EH38E1978366 | chr2:20220455-20220775 | dELS |
|  |  |  |  | chr2:20220618-20220632 | EH38E1978366 | chr2:20220455-20220775 | dELS |
|  |  |  | POU2AF1 | chr11:111375983-111375997 | EH38E1569180 | chr11:111375766-111376026 | dELS |
|  |  |  |  | chr11:111386927-111386941 | EH38E1569196 | chr11:111386665-111387013 | dELS |
|  |  |  |  | chr11:111387450-111387464 | EH38E1569197 | chr11:111387393-111387740 | dELS |
|  |  |  | PRDM1 | chr6:106076715-106076729 | EH38E2490424 | chr6:106076708-106076908 | dELS |
|  |  |  |  | chr6:106076768-106076782 | EH38E2490424 | chr6:106076708-106076908 | dELS |
|  |  |  |  | chr6:106080553-106080567 | EH38E2490428 | chr6:106080227-106080560 | dELS |
|  |  |  |  | chr6:106083601-106083615 | EH38E2490431 | chr6:106083606-106083935 | dELS |
|  |  |  |  | chr6:106086167-106086181 | EH38E2490437 | chr6:106086124-106086473 | PLS |
|  |  |  |  | chr6:106090289-106090303 | EH38E2490446 | chr6:106089989-106090307 | dELS |
| ATF6 | MA1466.1 | 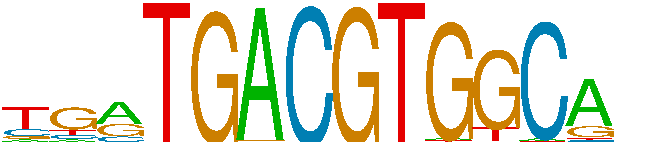 | MYC | chr8:127728986-127728999 | EH38E2666159 | chr8:127728876-127729167 | dELS |
|  |  |  |  | chr8:127735673-127735686 | EH38E2666168 | chr8:127735668-127735990 | pELS |
|  |  |  |  | chr8:127740535-127740548 | EH38E2666181 | chr8:127740428-127740731 | dELS |
|  |  |  |  | chr8:127743042-127743055 | EH38E2666187 | chr8:127742979-127743325 | dELS |
|  |  |  | POU2AF1 | chr11:111379326-111379339 | EH38E1569189 | chr11:111379181-111379530 | pELS |
| TF, transcription factor; TFBS, transcription factor binding sites; cCRE, candidate cis-regulatory elements; PLS. promoter-like signature; pELS, proximal enhancer-like signature; dELS, distal enhancer-like signature | | | | | | | |

**Supplementary Table S3. Pharmacokinetics data of NCP26**

| **Mean plasma concentration-time profiles of NCP26 in male C57BL/6 mice** | | | | | | | |
| --- | --- | --- | --- | --- | --- | --- | --- |
| **Summary of PK parameters** | T_max_ | C_max_ | T_1/2_ | MRT_last_ | MRT_inf_ | AUC_last_ | AUC_inf_ |
| X Code | (hr) | (ng/mL) | (hr) | (hr) | (hr) | (hr*ng/mL) | (hr*ng/mL) |
| NCP26  (PO-5 mg/kg) | 0.0830 | 327 | 2.29 | 0.797 | 1.00 | 188 | 192 |
| NCP26  (IP-1 mg/kg) | 0.0830 | 238 | 0.495 | 0.458 | 0.481 | 117 | 118 |

**Supplementary Table S4. Antibodies used in the study**

| **Western Blot (WB), Immunohistochemistry (IHC)** | | | |
| --- | --- | --- | --- |
| **Antibodies** | **Manufacturer** | **Catalog #** | **Applications** |
| EPRS | Abcam | ab31531 | WB |
| GCN2 | Abcam | ab134053 | WB |
| phopho-GCN2 | Abcam | ab75836 | WB |
| eIF2α | Cell signaling technology | 9722 | WB |
| phospho-eIF2α | Cell signaling technology | 9721 | WB |
|  | Abcam | 32157 | WB |
| ATF4 | Cell signaling technology | 11815 | WB |
| DDIT3 | Cell signaling technology | 2895 | WB |
| Cleaved Caspase-3 | Cell signaling technology | 9661 | WB |
|  | Cell signaling technology | 9664 | WB |
| Caspase-8 | Cell signaling technology | 9746 | WB |
| Caspase-9 | Cell signaling technology | 9502 | WB |
| PARP | Cell signaling technology | 9532 | WB |
|  | Cell signaling technology | 9542 | WB |
| MYC | Santa Cruz biotechnology | sc-40 | WB/IHC |
| PIM2 | Cell signaling technology | 4730 | WB |
| CCND1 | Cell signaling technology | 2922 | WB |
|  | Cell signaling technology | 55506 | IHC |
| CCND2 | Cell signaling technology | 3741 | WB |
| TCF3 | Santa Cruz biotechnology | sc-133075 | WB/IHC |
| PERK | Cell signaling technology | 5683 | WB |
| β-Actin-HRP | Cell signaling technology | 5125 | WB |
| GAPDH | Cell signaling technology | 2118 | WB |
| Α-Tubulin | Sigma | T6074 | WB |
| rabbit IgG-HRP | Cell signaling technology | 7074 | WB (2nd Ab) |
|  | Bethyl | A120-201P | WB (2nd Ab) |
| mouse IgG-HRP | Cell signaling technology | 7076 | WB (2nd Ab) |
|  | Santa Cruz | Sc-2005 | WB (2nd Ab) |
| Ki67 | Servicebio | GB13030-2 | IHC |
| **CyTOF** | | | |
| **Antibodies** | **Manufacturer** | **Catalogue#** | **Metal label** |
| CD45 | Fluidigm | 3089003B | Y89Di |
| IgGL | Biolegend | 316602 | In113Di |
| IgGK | Biolegend | 316502 | In115Di |
| CD81 | Biolegend | 349502 | Pr141Di |
| CD19 | Fluidigm | 3142001B | Nd142Di |
| Ikaros | Fluidigm | 3143024B | Nd143Di |
| CD38 | Fluidigm | 3144014B | Nd144Di |
| SLAMF7 | Biolegend | 331802 | Nd145Di |
| MCL-1 | Abcam | ab32087 | Nd146Di |
| CASP9 | Abcam | ab219590 | Sm147Di |
| BCL-XL | Abcam | ab199099 | Nd148Di |
| Bcl2 | Abcam | ab219608 | Sm149Di |
| P53 | Fluidigm | 3150024A | Nd150Di |
| IRF4 | Abcam | ab133590 | Eu151Di |
| Helios | Biolegend | 137202 | Sm152Di |
| CD267 | Biolegend | 311902 | Eu153Di |
| CASP8 | Abcam | ab32397 | Sm154Di |
| CD27 | Fluidigm | 3155001B | Gd155Di |
| PD-L1 | Fluidigm | 3156026B | Gd156Di |
| CD137L | Fluidigm | 3158022B | Gd158Di |
| CD138 | Biolegend | 356502 | Tb159Di |
| P16 | Abcam | ab108349 | Gd160Di |
| Ki67 | Fluidigm | 3161007B | Dy161Di |
| Aiolos | Fluidigm | 3162032B | Dy162Di |
| Bcl6 | Fluidigm | 3163012B | Dy163Di |
| CCND2 | Novus | NB100-1939 | Dy164Di |
| BCMA | Biolegend | 357502 | Ho165Di |
| CK1a | Life Technologies | PA571913 | Er166Di |
| Survivin | R&D systems | MAB886-SP | Er167Di |
| PLK1 | Abcam | ab17056 | Er168Di |
| FOXM1 | Invitrogen | 702664 | Tm169Di |
| CD117 | Biolegend | 313202 | Er170Di |
| CD307e | Biolegend | 340302 | Yb171Di |
| P21 | Abcam | ab80633 | Yb172Di |
| CD56 | Biolegend | 318345 | Yb173Di |
| ZFP91 | Celgene | Yb174Di |  |
| HDAC6 | Abcam | ab210472 | Lu175Di |
| MYC | Fluidigm | 3176012B | Yb176Di |

**Supplementary Table S5. Primers for qRT-PCR**

| Target genes | Directions | Sequenes |
| --- | --- | --- |
| EPRS | Forward | GTGGGCTCTCATCAAGTGG |
|  | Reverse | TTTTTGCCTCAAGACCCAAC |
| TCF3 | Forward | CATCTGCATCCTCCTTCTCC |
|  | Reverse | GAGTAGATCGAGGCCAGTGC |
| GAPDH | Forward | GAAGGTGAAGGTCGGAGTCA |
|  | Reverse | GGGGTCATTGATGGCAACAATA |

**Supplementary Table S6. shRNA vectors**

| Target genes | Vector name | Clone IDs | Target sequenes |
| --- | --- | --- | --- |
| EPRS | shEPRS #1 | TRCN0000293874 | ATGAACCTGTTAGCCCATATA |
|  | shEPRS #2 | TRCN0000293828 | CTCTTCAACTCCTCTCACTTT |
| GCN2 | shGCN2 | TRCN0000300851 | CCAAAGGTCTATCAAATGAAA |
| eIF2α | sheIF2α | TRCN0000344579 | GCAGTCTCAGACCCATCTATT |
| TCF3 | shTCF3 #1 | TRCN0000274218 | CCCGGATCACTCAAGCAATAA |
|  | shTCF3 #2 | TRCN0000017535 | CAGCCTCTCTTCATCCACATT |
| Luciferase | shLuc | TRCN0000072246 | CAAATCACAGAATCGTCGTAT |
